# Identification of tumor antigens and immune subtypes in head and neck squamous cell carcinoma for mRNA vaccine development

**DOI:** 10.3389/fcell.2022.1064754

**Published:** 2022-11-17

**Authors:** Yan Chen, Ning Jiang, Meihua Chen, Baiyan Sui, Xin Liu

**Affiliations:** ^1^ Department of Periodontology, Shanghai Stomatological Hospital & School of Stomatology, Fudan University; Shanghai Key Laboratory of Craniomaxillofacial Development and Diseases, Fudan University, Shanghai, China; ^2^ Department of Oral and Craniomaxillofacial Science, Shanghai Key Laboratory of Stomatology, College of Stomatology, Ninth People’s Hospital, Shanghai Jiao Tong University School of Medicine, Shanghai, China; ^3^ Department of Dental Materials, Shanghai Key Laboratory of Stomatology, Shanghai Biomaterials Research & Testing Center, Shanghai Ninth People’s Hospital, Shanghai Jiao Tong University School of Medicine; College of Stomatology, Shanghai Jiao Tong University; National Center for Stomatology; National Clinical Research Center for Oral Diseases, Shanghai, China

**Keywords:** head and neck squamous cell carcinoma, tumor antigens, immune subtypes, immune landscape, mRNA vaccine

## Abstract

The mRNA vaccines have been considered effective for combating cancer. However, the core components of the mRNA vaccines against head and neck squamous cell carcinoma (HNSCC) and the effects remain unclear. Our study aims to identify effective antigens in HNSCC to develop mRNA vaccines for corresponding potential patients. Here, we analyzed alternative splicing and mutation of genes in TCGA-HNSCC samples and identified seven potential tumor antigens, including SREBF1, LUC7L3, LAMA5, PCGF3, HNRNPH1, KLC4, and OFD1, which were associated with nonsense-mediated mRNA decay factor expression, overall survival prognosis and the infiltration of antigen-presenting cells. Furthermore, to select suitable patients for vaccination, immune subtypes related to HNSCC were identified by consensus clustering analysis, and visualization of the HNSCC immune landscape was performed by graph-learning-based dimensionality reduction. To address the heterogeneity of the population that is suitable for vaccination, plot cell trajectory and WGCNA were also utilized. HNSCC patients were classified into three prognostically relevant immune subtypes (Cluster 1, Cluster 2, and Cluster 3) possessing different molecular and cellular characteristics, immune modulators, and mutation statuses. Cluster 1 had an immune-activated phenotype and was associated with better survival, while Cluster 2 and Cluster 3 were immunologically cold and linked to increased tumor mutation burden. Therefore, HNSCC patients with immune subtypes Cluster 2 and Cluster 3 are potentially suitable for mRNA vaccination. Moreover, the prognostic module hub genes screened seven genes, including IGKC, IGHV3-15, IGLV1-40, IGLV1-51, IGLC3, IGLC2, and CD79A, which could be potential biomarkers to predict prognosis and identify suitable patients for mRNA vaccines. Our findings provide a theoretical basis for further research and the development of anti-HNSCC mRNA vaccines and the selection of suitable patients for vaccination.

## 1 Introduction

Head and neck squamous cell carcinoma (HNSCC) has been regarded as the most common histological type of head and neck malignancy and heterogeneous disease with a tendency for rapid recurrence and poor survival rates. According to GLOBOCAN data for 2020, the estimated incidence of HNSCC was approximately 930,000 cases and over 450,000 deaths (Sung et al., 2021). Genetic, smoking, drinking alcohol, and viral infections, such as Epstein–Barr virus (EBV) and human papillomavirus (HPV), are the most prevalent risk factors for HNSCC (Zhong et al., 2018; Gu et al., 2019). The primary treatment options include surgery combined with radiation therapy, chemotherapy, and targeted therapy. Despite the availability of combined modality treatment, HNSCC has been considered to have a poor prognosis, according to reports (Vermorken et al., 2014), and the estimated 5-year survival rate remains low (50%–55%) (Bray et al., 2018; Shen et al., 2020). The high mortality rate of HNSCC is due to resistance to therapy, driving local recurrences and distant metastases. Hence, there is an urgent need to develop different treatment patterns and prevention methods to improve the prognosis of HNSCC.

Immunotherapy, a strategy that boosts the patient’s immune system to treat malignancies, has recently emerged as a novel approach to overcome cancer, particularly immune checkpoint inhibitor (ICI) therapy, which has shown significant promise (Postow et al., 2018). Since 2016, two ICI therapies targeting relapsed and metastatic HNSCC, nivolumab and pembrolizumab, have been approved for marketing by the United States. Food and Drug Administration (FDA). However, only a fraction of patients currently benefit from ICI due to the stringent filtering indications (Ribas and Wolchok, 2018). Tumor vaccines have been another strategy attracting significant interest in immunotherapy as a result of their multifarious advantages, including increased specificity, ability to induce long-lasting immunity, effectively combating the low therapeutic efficacy, drug resistance, and side effects of conventional chemotherapy or ICI (Emens, 2006; Sayour et al., 2018). Prophylactic and therapeutic vaccines are the two primary categories for developing tumor vaccines. While prophylactic tumor vaccines can be used in healthy individuals to prevent cancer-causing infections, therapeutic tumor vaccines are usually used in advanced cancers to remove cancer cells (Bayó et al., 2021). In this research, we concentrate on therapeutic vaccines. Previous studies have confirmed that the therapeutic vaccines promote particular antitumor immune responses (Bayó et al., 2021; Faghfuri et al., 2021). Currently, tumor or immune cell vaccines, peptide vaccines, viral vector vaccines and DNA or RNA vaccines are the four primary categories of tumor vaccines (Faghfuri et al., 2021). mRNA vaccines represent a promising platform for tumor immunotherapy because of their safety, high potency, capacity for rapid development and potential for scalable manufacture (McNamara et al., 2015). mRNA vaccines stimulate a broader T-cell response (Van Nuffel et al., 2012), and multiple tumor-associated antigens (TAAs) or tumor-specific antigens (TSAs) are delivered, which also trigger cellular and humoral immune responses (Miao et al., 2021). In contrast to DNA vaccines, mRNA vaccines do not run the risk of insertional mutations by integration into the genome. As of now, mRNA tumor vaccines have demonstrated promise against melanoma, prostate, colorectal, and non-small-cell lung cancer (Kubler et al., 2015; Fiedler et al., 2016; Sullenger and Nair, 2016; Pardi et al., 2020; Shahnazari et al., 2020). However, no specific mRNA vaccines against HNSCC have been reported. Furthermore, considering the high level of tumor heterogeneity and the complicated tumor immune microenvironment, it is crucial to designate HNSCC patients that are suitable candidates for vaccination in order to maximize the efficacy and safety of vaccines.

In tumor cells, transcriptional regulation can be disrupted at various steps, leading to the accumulation of aberrant transcripts. However, aberrant transcripts can be subsequently degraded by nonsense-mediated mRNA decay (NMD) in the transcription of normal cells. Therefore, NMD has been considered a surveillance pathway used by cells to control the quality of mRNAs and to fine-tune transcript abundance (Kim et al., 2014; Baralle and Giudice, 2017; Zhang et al., 2021). Recent studies have shown that alternative splicing and NMD coupling could be a key posttranscriptional mechanism that regulates gene expression and has a significant impact on the transcriptome, and when NMD has been inhibited in tumor cells, frameshift mutations and aberrant splicing transcripts could produce neoantigen peptides (Goodman et al., 2017; Nogueira et al., 2021). Many biological processes, including proliferation, growth, differentiation and development, are controlled by alternative splicing (Kelemen et al., 2013). Protein isoforms produced by abnormal splicing can encourage the growth and development of tumors as well as resistance to treatment (Venables, 2004; David et al., 2010). An HNSCC patient’s alternative splicing signature can be used as a prognostic biomarker (Zhao et al., 2020). Additionally, neutral aberrant transcripts have the potential to be used as novel biomarkers for ICI therapy (Litchfield et al., 2020). For the current ICI therapy, tumor mutational burden (TMB) has been a potential biomarker positively correlated with its efficiency. TMB reflects cancer mutation quantity, and mutations are processed into neoantigens. Numerous pieces of evidence have revealed that tumors with a high mutation burden are related to greater infiltration of CD8^+^ T cells in tumor tissue, which could identify and eradicate these tumors. It suggests that TMB may influence the individual’s response to cancer immunotherapy (Rizvi et al., 2015; McGranahan et al., 2016). The evidence mentioned above is employed as the basis for our exploration of tumor antigens for the development of anti-HNSCC mRNA vaccines. At the same time, bioinformatics analysis is applied in this study, since it has been an essential tool for comprehending the molecular processes and signaling networks involved in cancer. Cancer diagnosis and treatment have advanced significantly as a result of the development of bioinformatics technology and the discovery of biomarkers (Bellairs et al., 2017; Leemans et al., 2018). Researchers may find tumor markers through the use of molecular-level data mining from various databases for clinical diagnosis or treatment (Chen and Coppola, 2018).

Herein, we aimed to use public data in the TCGA and GEO databases and apply bioinformatic analysis to identify potential tumor antigens of HNSCC. In addition, to identify patients with a higher vaccine response, multiple immune subtypes were discovered by consensus clustering analysis, and the cell components of different subtypes were characterized. Furthermore, to select suitable patients for vaccination, weighted gene co-expression network analysis was used to find prognostic gene modules and identify related genes to obtain the corresponding biomarker genes. Our findings will provide a reference for the development of anti-HNSCC mRNA vaccines.

## 2 Materials and methods

### 2.1 HNSCC patients

The Cancer Genome Atlas (TCGA) (https://portal.gdc.cancer.gov/) provided HNSCC patients for our research, which included 537 TCGA-HNSCC samples, of which 493 were cancer samples and 44 were normal samples (Supplementary Table S1). The RNA-seq data, Ensembl to Symbol annotation data, and clinical information of 493 HNSCC samples (Supplementary Table S2) were gathered from the University of California Santa Cruz (UCSC) Genome Browser (http://genome.ucsc.edu/). The Ensembl of RNA-seq data was converted to a gene symbol based on annotation data, and the gene lines annotated to the symbol were averaged and used as the new expression value of the gene. Next, the genes with zero expression in no less than 30% of the samples were removed, and samples that had a longer than 30-day survival duration were taken. Finally, samples of RNA-seq data, phenotypic data, and survival data were intersected. As an independent validation dataset, HNSCC samples for the GSE21122 dataset were gathered from the Gene Expression Omnibus (GEO) database (https://www.ncbi.nlm.nih.gov/gds/).

### 2.2 Screening of potential antigen candidate genes

The TCGASpliceSeq (https://bioinformatics.mdanderson.org/TCGASpliceSeq/) database provided HNSCC alternative splicing data. The percent spliced in (PSI) spectrum of alternative splicing events in TCGA-HNSCC was screened. The mean PSI values of alternative splicing events in cancer and normal samples were calculated separately and splicing events with mean values of 0 or 1 in samples were filtered out. A total of 40418 alternative splicing events were finally selected, and *t* tests and logFC calculations were performed. The BH method was used to correct the *p*-value of the *t* tests, and alternative splicing events with a false discovery rate (FDR) < 0.05 and | logFC | > 1 were treated as abnormal alternative splicing events.

Mutated genes were identified by mutation analysis in the Genomic Data Commons (GDC) (https://gdc.cancer.gov/) and cBioPortal (https://www.cbioportal.org/) databases. Using the R package “maftools”, mutated genes of TCGA-HNSCC samples were summarized and visualized.

Potential antigen candidate genes were chosen based on abnormal upregulation of alternative splicing events and frameshift mutations. Functional enrichment analysis was carried out using the R package “clusterProfiler.” The Kyoto Encyclopedia of Genes and Genomes (KEGG) pathway and Gene Ontology (GO) were used. GO team analysis was classified into three subgroups: biological process (BP), cellular component (CC), and molecular function (MF). Potential antigen candidate genes were matched with the genes from the RNA-seq expression profiles of TCGA-HNSCC, and then the differential expression of potential antigen candidate genes between cancer samples and normal samples was examined using the R package “limma”.

### 2.3 Identification of potential tumor antigens in HNSCC

The NMD factors included UPF1, UPF2, UPF3A and UPF3B, and these factors were divided into low and high expression groups according to their median expression levels in TCGA-HNSCC. The differences in the expression levels of potential antigen candidate genes between the two groups were analyzed. A *t* test was applied for analysis of variance with a threshold of *p* ≤ 0.05, and the R package was “ggpubr.” To recognize potential antigen candidate genes closely related to the overall survival prognosis, potential antigen candidate genes were analyzed using one-way Cox regression using the R packages “survival” and “survminer”, dividing the high and low expression groups by median gene expression values and analyzing the overall survival differences of the two groups, with a threshold of *p* < 0.05. Taking the intersection of potential antigen candidate genes that were differentially expressed in each NMD subgroup and potential antigen candidate genes with overall survival prognosis differences. To investigate and show the relationship between tumor immune infiltrating cells and intersection genes, we used the Tumor Immunization Estimation Resource (TIMER, https://cistrome.shinyapps.io/timer/) and *p* < 0.05 was regarded as statistically significant when using Spearman’s analysis.

### 2.4 Identification and verification of immune subtypes

Immune gene dataset was obtained from the Immport database (1255 genes) and the article “Pancancer immunogenomic analyses reveal genotype-immunophenotype relationships and predictors of response to checkpoint blockade” (782 genes). The final dataset with 1894 immune genes was obtained by taking the merged dataset. We used the intersection of the immune gene dataset and the genes in the RNA-seq data of TCGA-HNSCC and then screened immune genes related to overall survival prognosis. The final expression profile of 479 genes × 493 samples of the immune gene dataset in cancer samples was obtained. The R package “ConsensusClusterPlus” was used to perform consensus clustering of immune gene expression profiles.

To determine the repeatability of the immune subtypes, the same settings were used in the validation set to identify potential immune subtypes, and the final expression profiles contained 75 genes × 270 samples of the immune gene dataset in cancer samples.

### 2.5 Mutation status among immune subtypes

TMB data of HNSCC were obtained from the Genomic Data Commons (GDC) database (https://gdc.cancer.gov/). The TMB distribution among the subtypes was first demonstrated. Next, the mutation data of the corresponding samples were extracted by subtype, and the number of mutated genes in each subtype sample was counted. To display the mutations separately, the oncoplot function in the “maftools” package was used.

### 2.6 Immune modulators in different immune subtypes

The article “PMID33648511” provided immunogenic cell death (ICD) modulators and immune checkpoints (ICPs). The expression levels and differences of each factor between subtypes were presented. ANOVA was used with a threshold of *p* ≤ 0.05.

### 2.7 Cellular and molecular characteristics of immune subtypes

Using the R package “ESTIMATE”, the immune score, stromal score and tumor purity of cancer samples were computed. The distribution of these scores among different subtypes was shown using the R package “ggpubr.” Meanwhile, the heatmap and immune cell infiltration scores were displayed using “ComplexHeatmap”. The difference test was performed using the Wilcoxon test with a threshold of *p* ≤ 0.05, and the difference between groups was annotated on the heatmap using ANOVA with a threshold of *p* ≤ 0.05.

Pancancer immune subtypes of TCGA-HNSCC were obtained from the article “PMID29628290” and compared with the subtypes identified in this article. Differences in the proportion of pancancer immune subtypes were tested using ANOVA with a threshold of *p* ≤ 0.05.

### 2.8 Immune landscape of HNSCC

Utilizing the reduce Dimension function of the “Monocle” package with a Gaussian distribution, graph learning-based dimensionality reduction analysis was carried out to display the distribution of immunological subtypes across individual patients. The immune landscape of the cell trajectories of immune subtypes was shown in different colors. We calculated the Pearson correlation between principal components and cellular infiltration. The infiltration proportional differences between subtypes were determined using the Wilcoxon test with a threshold of *p* ≤ 0.05. Samples at extreme locations were chosen to demonstrate their survival.

### 2.9 WGCNA coexpression network construction

A coexpression network was constructed by weighted correlation network analysis (WGCNA) using the R package “WGCNA” for immune gene expression profiling. The number of genes in the resulting modules, as well as the ME scores of the samples in each module’s various subtypes were displayed. The prognostic module was identified using one-way Cox regression analysis with a threshold of *p* < 0.05. The prognosis-related module genes were identified using GO and KEGG enrichment analyses. The MM scores (correlation between genes and modules) of the prognostic module genes were calculated, and the hub genes of the prognostic module were chosen with an MM greater than 0.9. One-way Cox analysis was performed on hub genes, and the seven genes with the most significant Cox regression were screened as biomarker genes. A multifactorial Cox analysis of the seven biomarker genes was performed and the following equation was used to calculate a risk score using the coefficient value as a coefficient:

$${Riskscore}_{i}=\overset{n}{\underset{i=1}{\sum }}\exp_{ji}*\beta_{j},$$

Where exp_ji_ denoted the expression value of the j^th^ gene in the i^th^ sample and *β*_j_ denoted the coefficient of the j^th^ gene in the multifactorial Cox regression model. The median risk score was employed to classify high- and low-risk groups.

## 3 Results

### 3.1 Identification of potential antigen candidate genes

**FIGURE 1:**
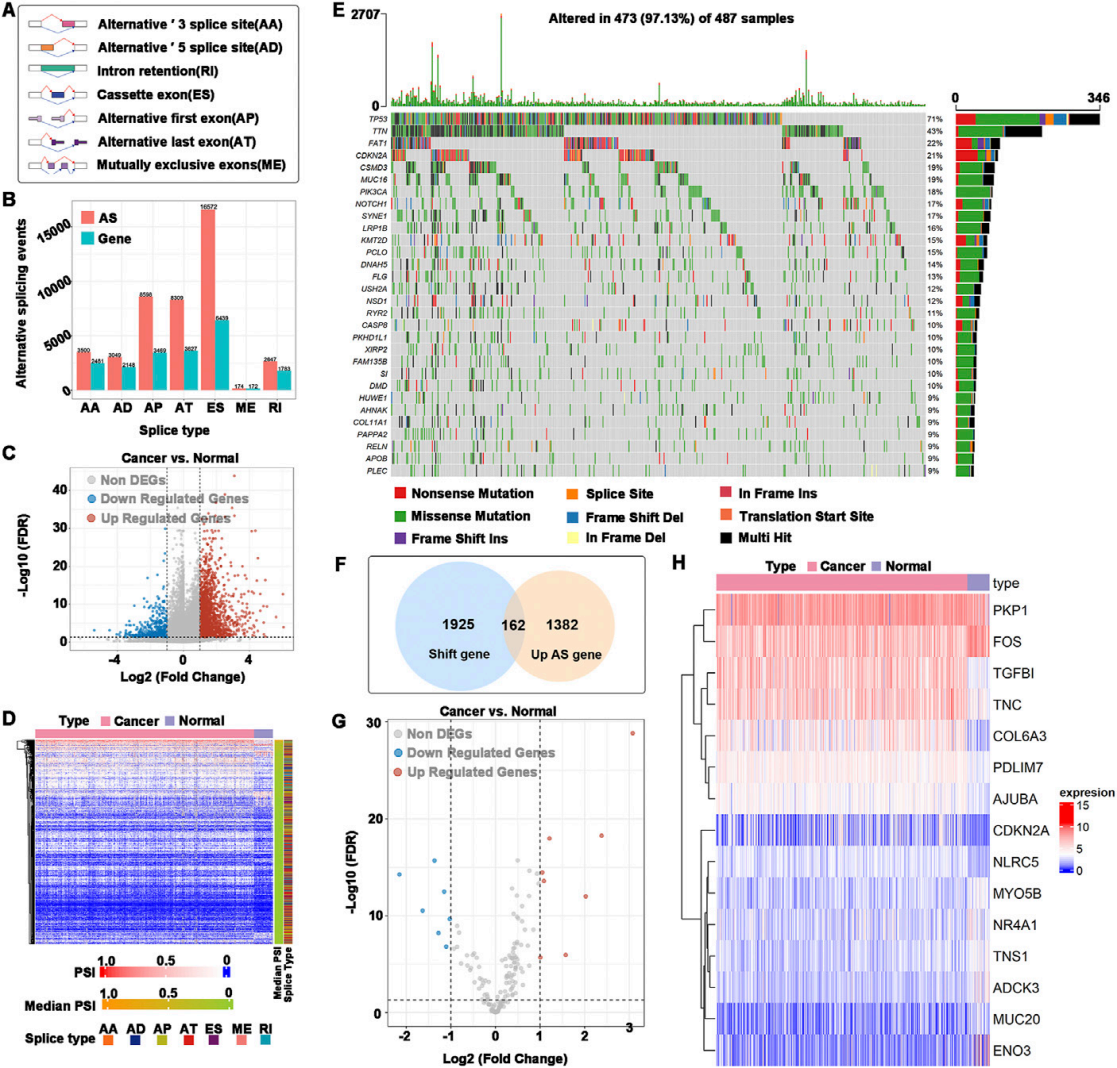
Identification of potential antigen candidate genes. **(A)** Types of alternative splicing events. **(B)** Numbers of alternative splicing events and corresponding genes in HNSCC. **(C)** Volcano plot of abnormal alternative splicing events. **(D)** Heatmap of abnormal alternative splicing events. **(E)** Waterfall chart of the mutated genes. Different colors represented different types of mutations. **(F)** Identification of potential antigen candidate genes. The intersection of abnormal upregulation of alternative splicing (1544 genes) and a frameshift mutation (2087 genes), of which 162 potential antigen candidate genes were identified. **(G)** Differentially expressed potential antigen candidate genes. **(H)** Heatmap of differentially expressed potential antigen candidate genes.

To find potential antigen candidate genes in HNSCC, an overview of 42849 alternative splicing events in TCGA-HNSCC was provided. The alternative splicing event that occurred the most was ES (16,572 events) and the least was ME (174 events) (Figures 1A,B). A total of 2476 abnormal alternative splicing events were filtered by *t* test and logFC calculation, which contained 1998 upregulated alternative splicing events (including 1544 genes) and 478 downregulated alternative splicing events (including 411 genes) (Figures 1C,D). Mutations include nonsense mutations, missense mutations, frame shift ins, splice sites, frame shift del, frame-del mutations, start site mutations (translation start site), and multiple coexisting mutations (multiple hits). The mutated genes in the samples were identified, and 2087 frameshift mutations were obtained. The top 30 genes with higher mutation probability were enriched in the waterfall chart, and different mutation types were represented by different colors (Figure 1E). The samples in altered genome fractions and mutation counts, representing a favorable immunogenic, which further demonstrates that the identified tumor antigens possess promising immunogenic (Supplementary Figures S1A,B). The upregulated genes generally may play an important role in tumorigenesis and tumor proliferation. The majority of tumor antigens come from upregulated genes and mutated genes (Coulie et al., 2014). Therefore, we intersected the genes with abnormal upregulation of alternative splicing (1544 genes) and frameshift mutations (2087 genes) as potential antigen candidate genes. A total of 162 potential antigen candidate genes were identified (Figure 1F). Functional enrichment analysis of 162 potential antigen candidate genes identified 20 functions of BP, 5 functions of CC and a KEGG pathway (Supplementary Figures S1C–E). The functions of BP were significantly enriched in cellular response to peptide, response to peptide hormone and aging. Focal adhesion and cell-substrate junction were highly enriched in the CC. The KEGG pathway was enriched in glycolysis gluconeogenesis. Potential antigen candidate genes matched to RNA-seq data of TCGA-HNSCC were 162 genes, and differential expression analysis identified 15 differentially expressed potential antigen candidate genes. As shown in Figures 1G,H, the up-regulated differentially expressed genes were TGFBI, COL6A3, PDLIM7, AJUBA, NLRC5, TNC, CDKN2A, and PKP1, as well as the down-regulated differentially expressed genes were ENO3, MUC20, ADCK3, TNS1, NR4A1, MYO5B, and FOS.

### 3.2 Identification of potential tumor antigens in HNSCC

**FIGURE 2:**
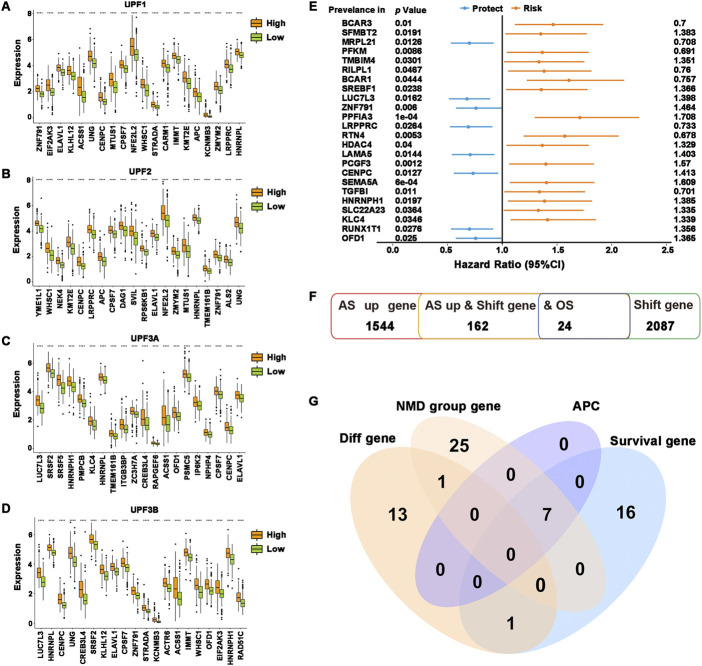
Identification of potential tumor antigens. **(A–D)** The top 20 most significantly differentially expressed genes were ranked according to the *p*-value of the *t*-test. **(A)** UPF1, **(B)** UPF2, **(C)** UPF3A, and **(D)** UPF3B. **(E)** Forest plot of overall survival prognosis-related genes. The first column was the genes, the second column was the *P*-*value* of Cox regression, and the last column was the HR value of the genes. **(F)** Overview of the number of abnormal upregulations of alternative splicing genes, frameshift mutation genes, and overall survival prognosis-related genes. **(G)** Venn diagram showing the overlapping genes among differentially expressed genes in potential antigen candidate genes differentially expressed genes in all NMD groups, APCs-related genes, and overall survival prognosis-related genes. ****p ≤ 0.0001.

To determine the relationship between NMD factors and potential antigenic candidate genes, the differential expression of 162 potential antigen candidate genes in the high- and low-expression groups of different NMD factors was demonstrated. The results showed that the expression levels of the top 20 differential potential antigen candidate genes in each group mostly coincided with NMD factor expression (Figures 2A–D). In addition, 1,064 alternative splicing events of 162 potential antigen candidate genes were obtained and analysis was performed on the differential distribution of their PSIs between the high- and low-expression groups of different NMD factors. The PSI values of the top 20 differential alternative splicing events were higher in the NMD factor high-expression group (Supplementary Figure S2). The quality-control mechanism of NMD might prevent tumor-causing cells from developing (Goodman et al., 2017; Nogueira et al., 2021). The results were consistent with the fact that when NMD factors were highly expressed, potential antigen candidate genes were also highly expressed to inhibit tumor progression. As previously stated, there is a close connection between NMD activity and alternative splicing. The results also confirmed that more differential alternative splicing events occurred in the NMD factor high-expression group. To obtain overall survival prognostic differences of potential antigen candidate genes, 24 genes associated with overall survival time in HNSCC were identified based on one-way Cox regression analysis, identifying the 8-protective factor indicators and 16-risk factor indicators (Figures 2E,F).

To identify potential tumor antigens in HNSCC, the intersection was taken between potential antigen candidate genes differentially expressed in each NMD group and potential antigen candidate genes with overall survival prognosis differences, and then the genes SREBF1, LUC7L3, LAMA5, PCGF3, HNRNPH1, KLC4, and OFD1 were obtained. Spearman analysis of the above genes and the ratio of immune infiltration showed that the expression of genes was significantly and positively correlated with tumor purity and the proportion of B cell, macrophage cell and dendritic cell infiltration (Supplementary Figure S3). According to the findings, these genes might be directly processed by antigen-presenting cells (APCs), presented to T cells, and then identified by B cells to initiate an immune response. Overall, SREBF1, LUC7L3, LAMA5, PCGF3, HNRNPH1, KLC4, and OFD1 were identified as possessing abnormal upregulation of alternative splicing, frameshift mutation, and NMD differential expression and were highly connected to both HNSCC prognosis and the infiltration of APCs (Figure 2G), which exhibit excellent properties of tumor antigens for the development of anti-HNSCC mRNA vaccines.

### 3.3 Identification of immune subtypes

**FIGURE 3:**
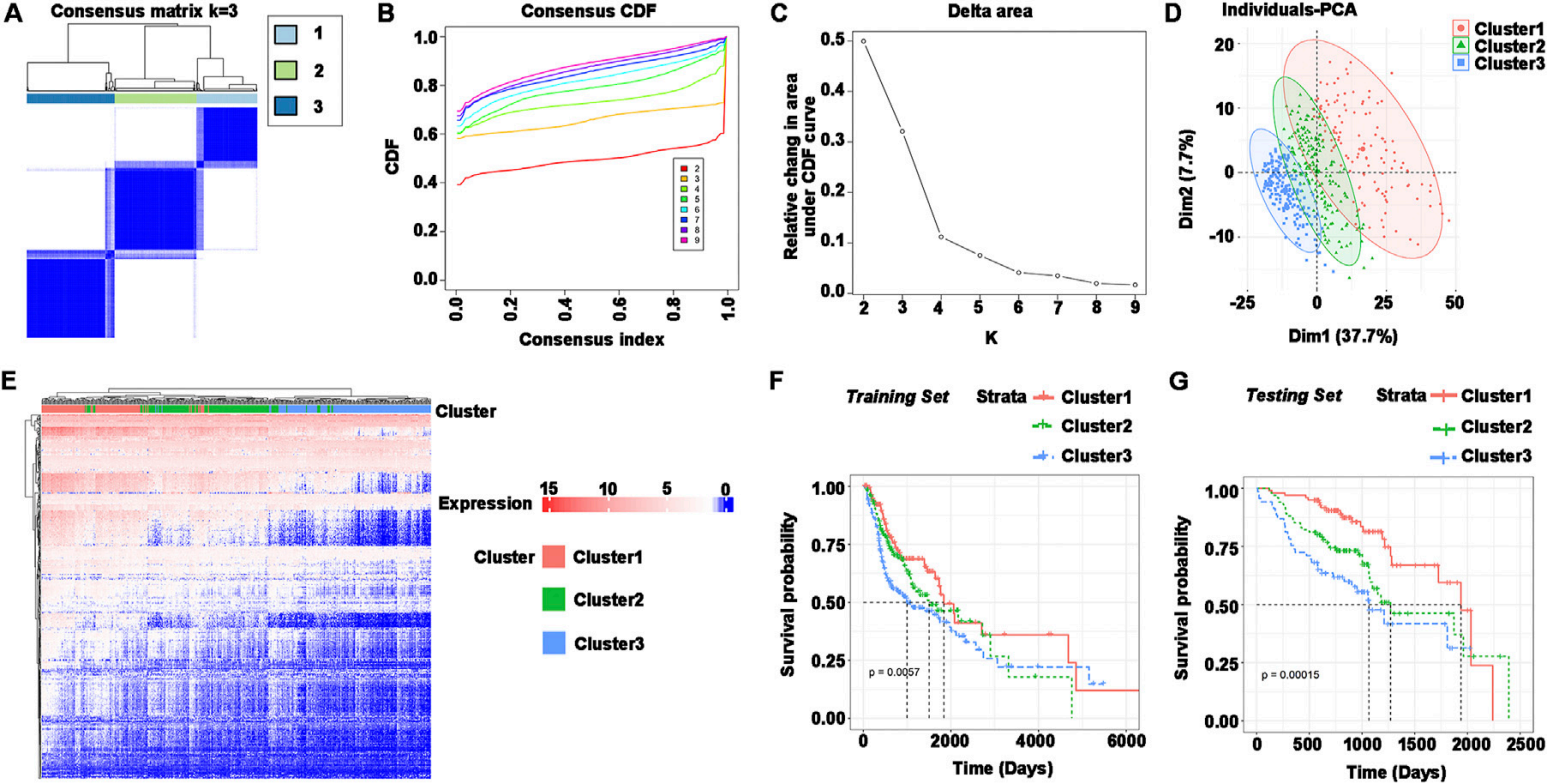
Identification of immune subtypes in HNSCC. **(A–C) (A)** Consensus matrix heatmap, **(B)** cumulative distribution function curve, and **(C)** delta area curve based on immune-related gene expression profile. **(D)** Distribution of principal component analysis. **(E)** Heatmap of immune gene set expression in Cluster1, Cluster2, and Cluster3. Genes and samples were clustered separately. **(F)** Survival curves for Cluster1, Cluster2, and Cluster3 in the training set (TCGA). **(G)** Survival curves for Cluster1, Cluster2, and Cluster3 in the testing set (GEO).

The immune subtypes have been considered to be reflecting the immune state and microenvironment of the tumor, contributing to the identification of suitable vaccinated patients. Consensus clustering was created when the expression profiles of prognosis-related genes in HNSCC samples were evaluated. k = 3 was chosen for stable clustering of immune-related genes based on their accumulative distribution functions and incremental regions, and three immune subtypes were obtained, given the names Cluster 1, Cluster 2, and Cluster 3 (Figures 3A–C). Principal component analysis was used to validate the three subtypes, and the findings demonstrated that they could be easily identified (Figure 3D). Genes and samples were clustered separately to illustrate the expression of immune genes in three subtypes (Figure 3E). The survival curves demonstrated that the overall prognosis of Cluster 1, Cluster 2, and Cluster 3 were significantly different. Cluster 1 had a better prognosis, whereas Cluster 3 had the lowest chance of survival (Figure 3F). The validation set was similarly clustered, and there was a significant difference in overall survival between the three subtypes (Figure 3G). Taken together, immune subtypes can be utilized to forecast the prognosis of HNSCC patients.

### 3.4 Mutation status among immune subtypes

**FIGURE 4:**
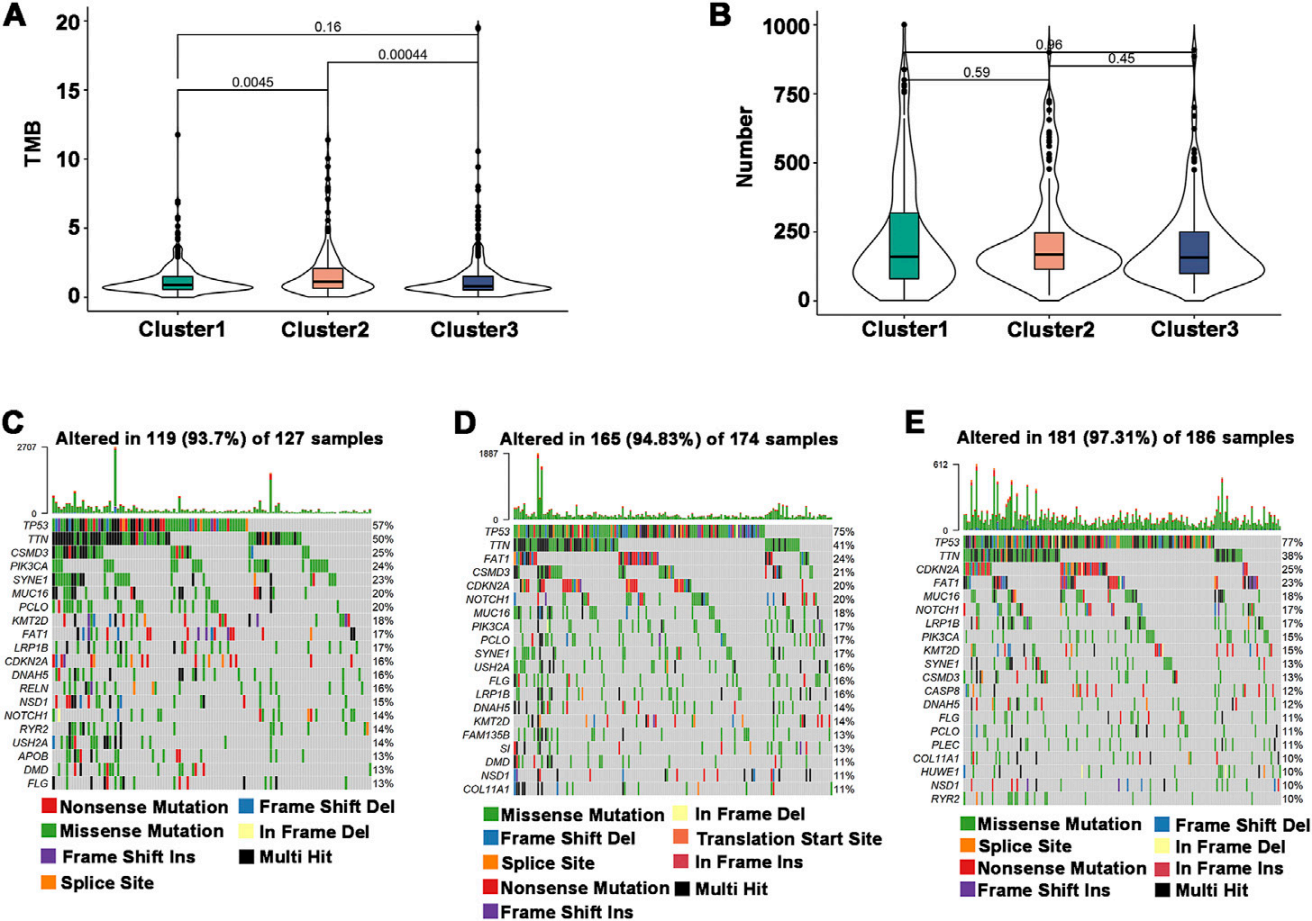
Association of immune subtypes with TMB and mutation. **(A)** TMB of different immune subtypes. **(B)** Mutation number of different immune subtypes. **(C–E)** Waterfall diagram of the top 20 frequently mutated genes in the three immune subtypes. **(C)** Cluster 1, **(D)** Cluster 2, and **(E)** Cluster 3.

The efficiency of tumor immunotherapy is highly correlated with TMB and the quantity of mutations in tumor patients (Sha et al., 2020). Herein, using the mutation dataset acquired from TCGA-HNSCC, TMB and the number of mutations in individual patients in different subtypes were evaluated. The difference in TMB score between Cluster 1 and Cluster 3 was not significant, and Cluster 2 had the highest TMB score (Figure 4A). The number of mutations between subtypes was not significantly different (Figure 4B), but the highest mutation rate was 97.31% in Cluster 3, followed by Cluster 2 (94.83%) and Cluster 1 (93.7%) (Figures 4C–E). In addition, the most frequently mutated genes in HNSCC subtypes were TP53 and TTN. These findings indicated that Cluster 1 may respond to immunotherapy less favorably than Cluster 2 and Cluster 3.

### 3.5 Immune modulators in different immune subtypes

**FIGURE 5:**
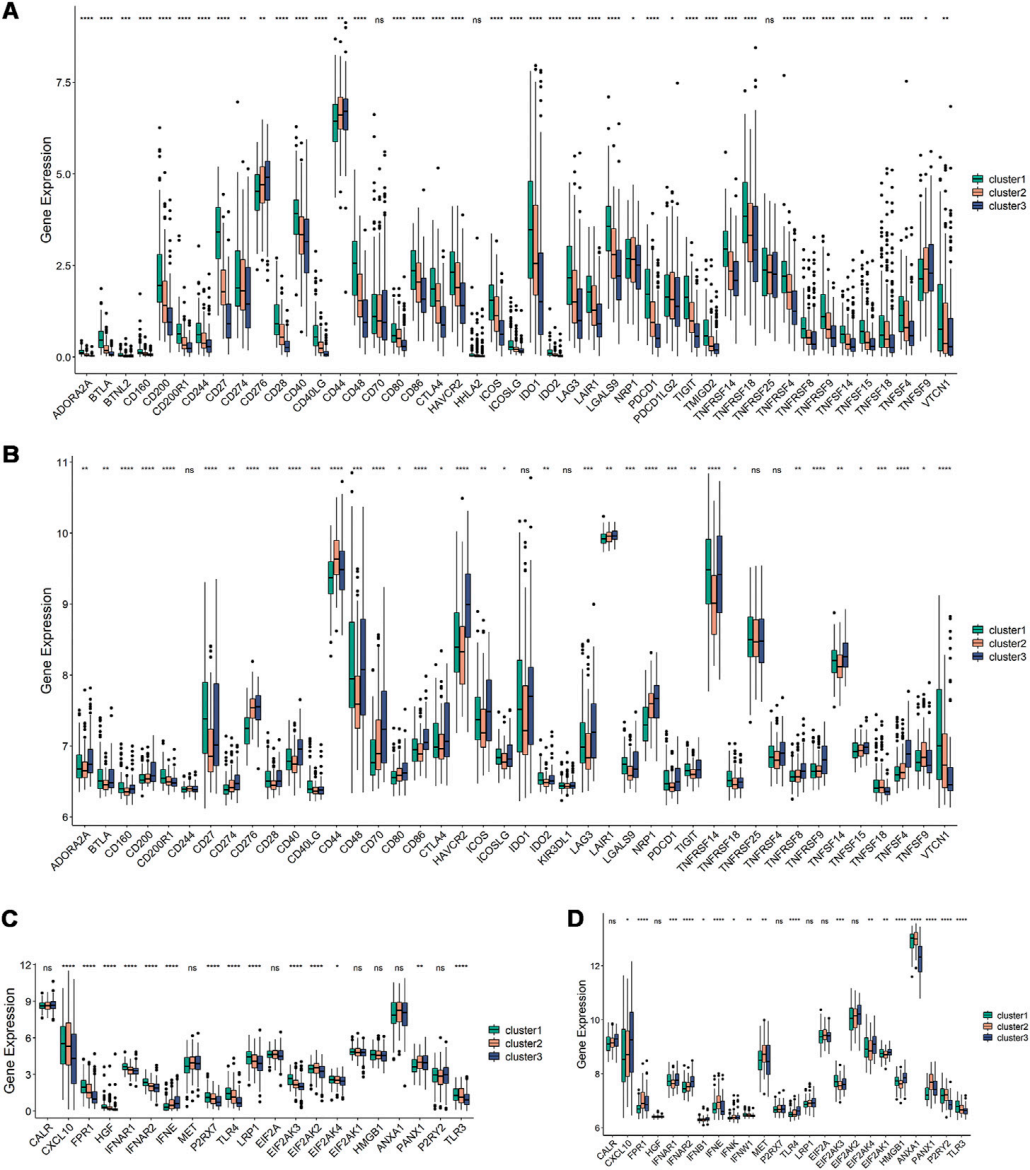
Relationships among ICPs and ICD modulators and the immune subtypes. **(A,B)** Distribution of ICP genes among the three immune subtypes in the TCGA and GEO dataset. **(C,D)** Distribution of ICD genes among the three immune subtypes in the TCGA and GEO dataset. *p ≤ 0.05, **p ≤ 0.01, ***p ≤ 0.001, ****p ≤ 0.0001, and ns, non-significant.

The efficiency of mRNA vaccines may be assessed using ICPs and ICD modulators, which are crucial in cancer immunity (Hodges et al., 2017; Galluzzi et al., 2020). Therefore, the expression levels of ICP- and ICD-related genes in different immune subtypes were analyzed. The results showed that ICP-related genes were differentially expressed between the immune subtypes in the TCGA dataset (93.75%) and validation set (88.1%) (Figures 5A,B). For instance, most ICP-related genes, including CD200, CD27, CD28, CD40, CTLA4, HAVCR2, IDO1, IDO2, LGALS9, TNFRSF9, and VTCN1, were elevated in Cluster 1 in the TCGA dataset, while they were expressed at lower levels in Cluster 2 and Cluster 3. CD27, CD40, CD48, CTLA4, TIGIT, TNFRSF14, and TNFSF14 were significantly upregulated in Cluster 1 and Cluster 3 in the validation dataset. There were 14 (66.67%) and 18 (75%) ICD-related genes that showed differences in the TCGA dataset and validation set, respectively (Figures 5C,D). For example, CXCL10, EIF2AK2, and TLR3 were significantly increased in Cluster 2 in the TCGA dataset, while FPR1, IFNE, MET, ANXA1, and PANX1 were elevated in Cluster 2 in the validation set. Thus, the immune subtypes may serve as potential therapeutic indicators for mRNA vaccines by reflecting the expression levels of ICPs and ICD modulators. Given the high expression of ICP-associated genes in Cluster 1, which has immunosuppressive tumor microenvironment and might not be appropriate for developing mRNA vaccines.

### 3.6 Cellular and molecular characteristics of immune subtypes

**FIGURE 6:**
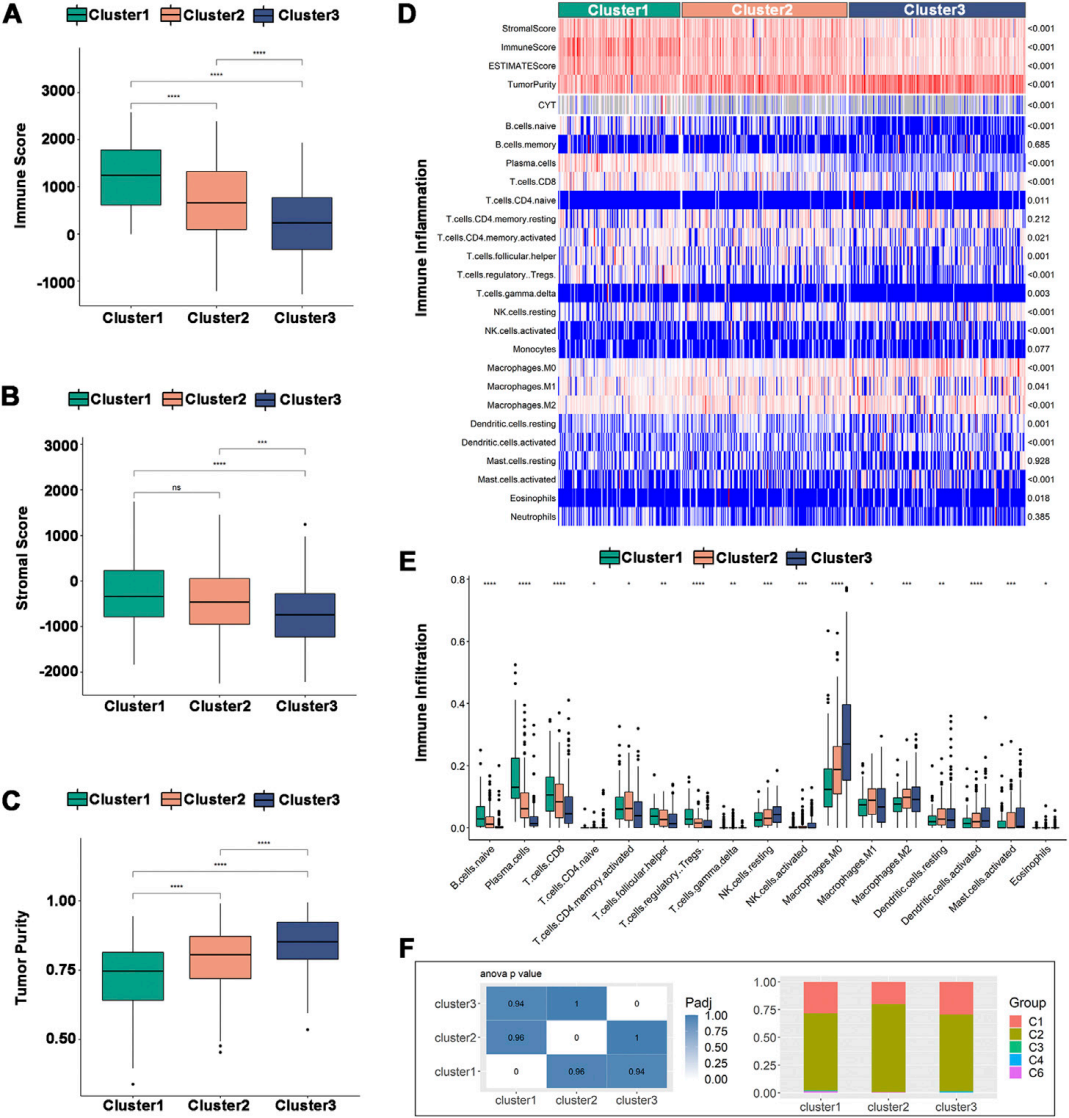
Cellular and molecular characteristics of immune subtypes in TCGA. **(A–C)** Differential distribution of **(A)** immune scores, **(B)** stromal scores, and **(C)** tumor purity among the three immune subtypes. **(D)** Heatmap of immune cell infiltration ratio. **(E)** Differential distribution of immune cell infiltration ratio. **(F)** Distribution of Cluster1-Cluster3 between pan-cancer immune types (C1-C4, C6). *p ≤ 0.05, **p ≤ 0.01, ***p ≤ 0.001, ****p ≤ 0.0001, and ns, non-significant.

Tumor purity can be predicted by the immune score and stromal score, which can also be used to estimate the immune cell and stromal cell content in tumors. High stromal cell and immune cell contents are associated with low tumor purity and *vice versa*. In the TCGA dataset, Cluster 1 had a higher immune score and stromal score (Figures 6A,B) but a lower tumor purity (Figure 6C). The immune cell infiltration ratio heatmap (Figure 6D) and distribution of differences (Figure 6E) showed that 17 immune cells had intergroup variations. Plasma cells, CD8^+^ T cells, regulatory T cells and follicular helper T cells had larger immune cell infiltration ratios in Cluster 1, whereas NK cells, macrophages, dendritic cells, and activated mast cells had higher immune cell infiltration ratios in Cluster 2 and Cluster 3. In the validation set, Cluster 3 had a lower tumor purity (Supplementary Figures S4A–C), and there was intergroup variability in the immune cell infiltration ratio of 12 immune cells. The immune cell infiltration ratios of plasma cells, CD8^+^ T cells, activated CD8^+^ T cells, follicular helper T cells, regulatory T cells and gamma-delta T cells were higher in Cluster 1, while monocytes, M0 macrophages, activated mast cells and neutrophils were significantly higher in Cluster 2 and Cluster 3 (Supplementary Figures S4D, E). Therefore, Cluster 2 and Cluster 3 were higher compared to Cluster 1 on anti-tumor immune cells, such as activated dendritic cells and NK cells, as well as pro-tumor immunosuppressive cells, such as tumor-associated macrophages. The results indicated that all three immune subtypes had complex tumor immune microenvironments. Cluster 1 was an immune-activated and immunologically hot phenotype, while Cluster 2 and Cluster 3 were immunologically cold phenotypes. The immune subtypes are available to support the identification of patients benefiting from mRNA vaccination and may be a reflection of the immune status for HNSCC.

The correlation between the immune subtypes and pancancer immune subtypes was explored to support the validity of the immune subtypes. In the six pancancer immune subtypes (C1-C6) previously reported, HNSCC was mostly clustered into C1 (wound healing) and C2 (IFN-γ dominant) and very little clustered into C3 (inflammatory), C4 (lymphocyte depleted) and C6 (TNF-β dominant) (Thorsson et al., 2018). Our results were consistent with previous results and showed more clusters in C2 than in C1. Additionally, there was more overlap with C2 in Cluster 2, implying that Cluster 2 may be more related to C2 than Cluster 1 and Cluster 3 (Figure 6F).

### 3.7 Immune landscape of HNSCC

**FIGURE 7:**
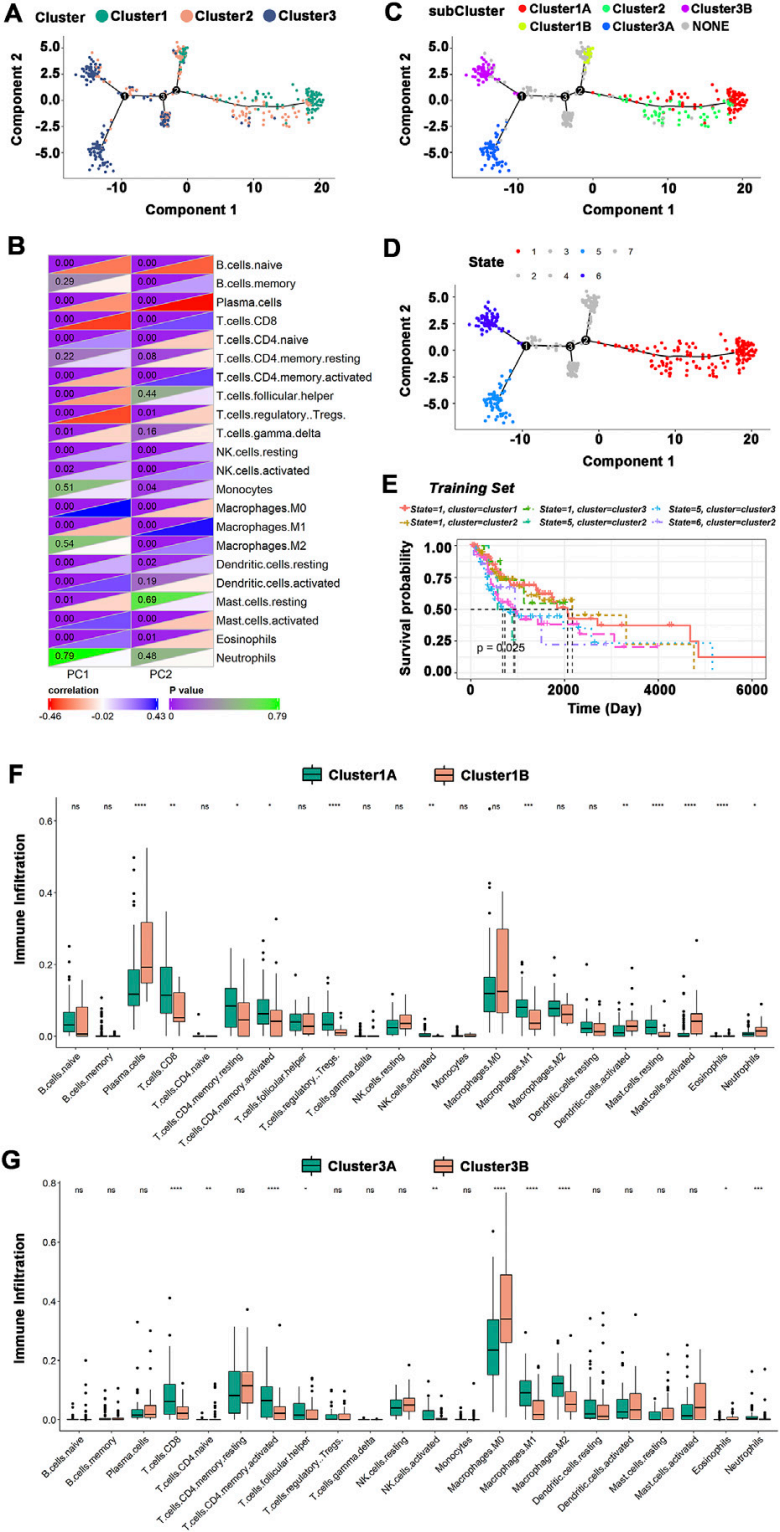
The immune landscape of HNSCC. **(A)**The immune landscape of HNSCC immune subtypes. Each dot represented a patient, and different colors represented different immune subtypes. The horizontal axis represented the principal component 1, and the vertical axis represented the principal component 2. **(B)** Heatmap of the correlation between principal components 1 and 2 and immune cells. **(C)** The immune landscape of the subgroups of HNSCC immune subtypes. **(D)** The immune landscape of samples from extreme distributional positions. **(E)** Prognosis of extreme distributional places. **(F,G)** Differences in enrichment scores of immune cells among subgroups: Cluster 1 **(F)** and Cluster 3 **(G)**. *p ≤ 0.05, **p ≤ 0.01, ***p ≤ 0.001, ****p ≤ 0.0001, and ns, non-significant.

The immune landscape of HNSCC was constructed using the immune gene expression profiles of individual patients (Figure 7A), and HNSCC patients were divided into different clusters. B cells, T cells, macrophages, etc., were significantly correlated with the first principal component (PC1) and the second principal component (PC2) (Figure 7B). The integral distribution of Cluster 1 was opposite to that of Cluster 3, and the distributions of the two subtypes were discrete. In addition, the same subtype additionally showed an opposite distribution, revealing strong intracluster variation within subtypes, particularly within Cluster 2. Cluster 1 and Cluster 3 were each further separated into two subgroups based on the distribution of immune cell populations (Figure 7C), and the enrichment scores of several immune cells varied dramatically between subtypes (Figures 7F,G). Cluster 1A had significantly higher enrichment scores for CD8^+^ T cells, resting memory CD4^+^ T cells, activated memory CD4^+^ T cells, regulatory T cells, M1 macrophages, and resting mast cells, while Cluster 1B had significantly higher enrichment scores for plasma cells, activated dendritic cells, activated mast cells, and neutrophils. CD8^+^ T cells, activated memory CD4^+^ T cells, follicular helper T cells, activated NK cells, M1 macrophages, and M2 macrophages had considerably higher enrichment scores in Cluster 3A, while Cluster 3B had significantly higher enrichment scores in M0 macrophages. Furthermore, prognostic comparisons of samples with extreme distributional positions in the immune landscape revealed that patients in Cluster 1 had the best survival probability (Figures 7D,E). Taken as a whole, the immune landscape based on immune subtypes can accurately identify the immunological components of each HNSCC patient and predict their prognoses, which is advantageous for choosing customized therapies for mRNA vaccination.

### 3.8 WGCNA coexpression network construction and hub genes

**FIGURE 8:**
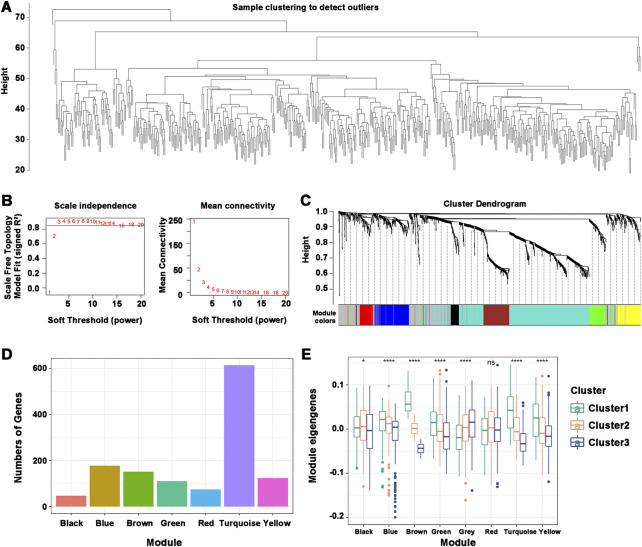
Characterization of the immune gene coexpression module of HNSCC. **(A)** Sample Clustering. **(B)** Scale-free fit index (β) and mean connectivity for every soft threshold power. **(C)** Tree diagram of all immune-related genes clustered based on the TOM matrix. **(D)** Number of genes in every module. **(E)** Differential distribution of module eigenvectors in HNSCC immune subtypes. *p ≤ 0.05, ****p ≤ 0.0001, and ns, non-significant.

Understanding the characteristics of each immune subtype was aided by identifying the functional modules of immune-related genes in HNSCC patients. The data were grouped by the immune gene coexpression module using WGCNA and a soft threshold of 3 for the scale-free network (Figures 8A,B). The representation matrix was then transformed into an adjacency matrix, which was then transformed into a topological matrix. Using the default parameters of the “WGCNA” R package, we obtained 8 modules, of which the gray modules were not clustered with the rest (Figure 8C). The number of genes in each module was shown, and an in-depth analysis of the distribution of immune subtypes in the eight modules was provided. Significant differences were found in all modules, except for the red module (Figures 8D,E). Brown, turquoise, and yellow modules had the most eigengenes in Cluster 1. The number of eigengenes in Cluster 2 was significantly the highest in the Black and Green modules, while that in Cluster 3 was Gray and Black. Thus, Cluster 1 corresponded to immunologically hot phenotypes, and Cluster 2 and Cluster 3 corresponded to immunologically cold phenotypes. Further prognostic analysis of the identified modules revealed significant prognostic efficacy for the Turquoise and Brown modules (Supplementary Figure S5A). To investigate the functions of genes in the Turquoise and Brown modules, a KEGG functional enrichment analysis was performed, and the first ten pathways were displayed (Supplementary Figures S5B, C). The immune-related cytokine-cytokine receptor interaction pathway was enriched in both modules. Genes with Turquoise and Brown module correlations >90% were selected as the hub genes of the prognostic module, and a total of 77 genes were identified. Seven marker genes, including IGKC, IGHV3-15, IGLV1-40, IGLV1-51, IGLC3, IGLC2, and CD79A, were screened by one-way Cox regression as potential biomarkers.

The risk model was used to score the seven biomarker genes. Samples with scores above the median were defined as the high-risk group, and samples with scores below the median were defined as the low-risk group (Supplementary Figure S6A). The low-risk group had a better survival status than the high-risk group (Supplementary Figures S6B, C). Prognostic analysis of the risk score was performed to demonstrate its prognostic efficacy, and the area under the curve (AUC) was used to verify its accuracy. The AUC curves of the risk score were 0.653, 0.622, and 0.575 at 1, 3, and 5 years, respectively (Supplementary Figure S6D), verifying their accuracy and indicating that they have potential as biomarkers for mRNA vaccines of HNSCC. The expression heatmap showed that all seven biomarker genes were highly expressed in the low-risk group (Supplementary Figure S6E). In summary, HNSCC patients with higher expression of IGKC, IGHV3-15, IGLV1-40, IGLV1-51, IGLC3, IGLC2, and CD79A may present with a more favorable prognosis, which are available to forecast prognosis and choose patients suitable for mRNA vaccines.

## 4 Discussion

The trending subject in cancer immunotherapy is currently the development of mRNA vaccines (Huang et al., 2021a; Huang et al., 2021b; Ye et al., 2021; Zhong et al., 2021). In our study, we identified a series of potential tumor antigens, including SREBF1, LUC7L3, LAMA5, PCGF3, HNRNPH1, KLC4, and OFD1, by systematically analyzing alternative splicing and mutation of genes in patients with HNSCC. They were associated with not only NMD expression and overall survival prognosis but also the infiltration of APCs. As a result, we deduce that these potential tumor antigens are crucial for the initiation and development of HNSCC and have the ability to cause an immunological attack by inducing a powerful cytolytic CD8^+^ T cell response. Previous investigations confirmed their potential for the creation of anti-HNSCC mRNA vaccines, even if functional validation and preclinical evaluation are still required in the future. For example, SREBF1 is essential for squamous cell carcinoma viability and migration, and its overexpression is associated with poor survival in squamous cell carcinoma patients. Squamous cell carcinomas may have SREBF1 as a potential therapeutic target and prognostic marker (Li et al., 2021). LUC7L3 is an mRNA that has rarely been studied for its biological role in disease. Only the fact that LUC7L3 is increased in human heart failure tissues and exacerbates heart failure is known (Gao et al., 2011). In many types of cancer, LAMA5 has been observed to be highly expressed and is a possible candidate for targeting angiogenesis in cancer (Hao et al., 2001). In addition, the PCGF3 gene, which is a member of the polycombgroup of proteins, has been shown to be involved in deregulating proteins that could lead to cancer cell transformation (Valk-Lingbeek et al., 2004). HNRNPH1, belonging to the heterogeneous nuclear ribonucleoprotein family, is an RNA binding protein that is involved in pre-mRNA splicing and mRNA trafficking and stability (Turunen et al., 2013; Grammatikakis et al., 2016). HNRNPH1 is associated with survival, and its high expression is associated with poor outcome in HNSCC (Xing et al., 2019). Moreover, KLC4 is a light chain isoform of kinesin that is observed at higher levels in tumor tissue than in healthy tissue. It is a brand-new and powerful cancer therapeutic marker, particularly for individuals with radiation resistance (Baek et al., 2018). One important element, OFD1, is employed to prevent primary cilia in human cancer cells from growing (Huang et al., 2015). In patients with oropharyngeal squamous cell carcinoma, HPV may regulate OFD1 expression and primary cilia formation, thereby influencing tumor progression and making this protein a possible target for treatment (Meng et al., 2020). These potential tumor antigens can be used as a basis for the subsequent search for identified tumor antigens in the development of mRNA vaccines in the future.

The selection of vaccinable patients is crucial for the efficacy of HNSCC mRNA vaccines. Immune subtypes can aid in discovering suitable vaccinated patients by reflecting the immune state and tumor microenvironment. Therefore, we classified the HNSCC samples into three different subgroups based on the gene expression profiles related to immunological prognosis. The three immune subtypes exhibited different molecular and cellular characteristics. They can be used to identify the prognosis of HNSCC patients, as Cluster 1 was linked to a better prognosis, while Cluster 3 had the worst survival probability. In addition, Cluster 2 had a relatively higher TMB, and Cluster 3 had the highest somatic mutation rates. According to the pertinent literature, immune status may be connected to mutation (Leemans et al., 2018). High somatic mutations and TMB were linked to a higher antitumor immune response (Rooney et al., 2015). Therefore, compared to Cluster 2 and Cluster 3, Cluster 1 may have a lower response to immunotherapy. Moreover, Cluster 1 had relatively higher expression of ICPs, suggesting that Cluster 1 might respond well to passive immunotherapies, such as blocking therapy; however, it might not be suitable for anti-HNSCC mRNA vaccines. It is well known that the tumor immune status determines the efficacy of mRNA vaccines. Thus, immune and stromal cell scores in different subtypes were analyzed to predict tumor purity and immune cell components. In the TCGA dataset, Cluster 1 had a lower tumor purity and an immune cell infiltration ratio that was significantly higher for plasma cells, CD8^+^ T cells, regulatory T cells and follicular helper T cells. Cluster 2 and Cluster 3 had higher immune cell infiltration ratios of NK cells, macrophages, dendritic cells, and activated mast cells. However, in the validation set, Cluster 2 and Cluster 3 had a lower tumor purity. The immune cell infiltration ratios of plasma cells, CD8^+^ T cells, activated CD8^+^ T cells, follicular helper T cells, regulatory T cells and gamma-delta T cells were higher in Cluster 1, while monocytes, M0 macrophages, activated mast cells and neutrophils were significantly higher in Cluster 2 and Cluster 3. We inferred from the results that Cluster 1 was an immune-activated and immunologically hot phenotype, while Cluster 2 and Cluster 3 were immunologically cold phenotypes. Furthermore, immune cell infiltration and the cancer microenvironment have both been relevant to cancer prognosis. Previous studies have shown that naive B cells were immune cells that fight cancers (Katsuta et al., 2019). CD8^+^ T cells were associated with better prognosis in a number of cancer types (Sato et al., 2005). Meanwhile, a better prognosis was linked to greater B/plasma cell and T cell infiltration (Kurebayashi et al., 2018), whereas macrophages had been linked to a worse prognosis (Edin et al., 2012; Noy and Pollard, 2014). These molecular characteristics and the immune profiles were identical, indicating that HNSCC patients with different immune subtypes have dramatically varying specificity for mRNA vaccines. Finally, the complex immune landscape of HNSCC indicated that there was much variation across patients and even within the same immune subtype, which may significantly limit the effectiveness of mRNA vaccines. IGKC, IGHV3-15, IGLV1-40, IGLV1-51, IGLC3, IGLC2, and CD79A were identified as hub genes, and a validated prognostic risk model was constructed based on the seven genes and created two groups with different levels of risk for the samples. All seven biomarker genes were strongly expressed in samples from the low-risk group, which also had a higher survival status. These results implied that the potential biomarkers IGKC, IGHV3-15, IGLV1-40, IGLV1-51, IGLC3, IGLC2, and CD79A may be utilized to forecast prognosis and choose patients suitable for mRNA vaccines.

The HNSCC patients were categorized into C1-C6 subtypes, with the exception of C5, based on a prior study (Thorsson et al., 2018), and the majority of patients were grouped into the C1 and C2 subtypes. In the present study, HNSCC patients were differentiated into three subtypes, and all mainly overlapped with C1 and C2, which was consistent with previous findings. The results indicated that our immunotyping method was reliable. Nevertheless, the vaccination tumor antigens and prognostic indicators discovered in this study still need to be verified in subsequent research.

## 5 Conclusion

We used public data in the TCGA and GEO databases and identified the prospective HNSCC tumor antigens for the development of mRNA vaccines, including SREBF1, LUC7L3, LAMA5, PCGF3, HNRNPH1, KLC4, and OFD1, which are associated with NMD factor expression, overall survival prognosis and infiltration of APCs. The immune subtypes obtained by consensus clustering analysis and the results of a series of characterization proved that the immune subtypes Cluster 2 and Cluster 3 in HNSCC are most likely to respond well to mRNA vaccination. Meanwhile, prognostic module genes identified by WGCNA, including IGKC, IGHV3-15, IGLV1-40, IGLV1-51, IGLC3, IGLC2, and CD79A, could be potential biomarkers for predicting prognosis and identifying individuals that would benefit from mRNA vaccinations. Our findings provide a theoretical foundation for further research, including the development of anti-HNSCC mRNA vaccines and the identification of appropriate patients for vaccination.

## Data Availability

The datasets presented in this study can be found in online repositories. The names of the repository/repositories and accession number(s) can be found in the article/Supplementary Material.

## References

[B1] Baek J. H. Lee J. Yun H. S. Lee C. W. Song J. Y. Um H. D. (2018). Kinesin light chain-4 depletion induces apoptosis of radioresistant cancer cells by mitochondrial dysfunction via calcium ion influx. Cell Death Dis. 9, 496. 10.1038/s41419-018-0549-2 29717133 PMC5931584

[B2] Baralle F. E. Giudice J. J. N. R. M. C. B. (2017). Alternative splicing as a regulator of development and tissue identity. Nat. Rev. Mol. Cell Biol. 18, 437–451. 10.1038/nrm.2017.27 28488700 PMC6839889

[B3] Bayó C. Jung G. Español-Rego M. Balaguer F. Benitez-Ribas D. J. I. J. O. M. S. (2021). Vaccines for non-viral cancer prevention. Int. J. Mol. Sci. 22, 10900. 10.3390/ijms222010900 34681560 PMC8535337

[B4] Bellairs J. A. Hasina R. Agrawal N. J. C. Reviews M. (2017). Tumor DNA: An emerging biomarker in head and neck cancer. Cancer Metastasis Rev. 36, 515–523. 10.1007/s10555-017-9685-x 28801876 PMC5839661

[B5] Bray F. Ferlay J. Soerjomataram I. Siegel R. L. Torre L. A. Jemal A. (2018). Global cancer statistics 2018: GLOBOCAN estimates of incidence and mortality worldwide for 36 cancers in 185 countries. Ca. Cancer J. Clin. 68, 394–424. 10.3322/caac.21492 30207593

[B6] Chen J. Coppola G. (2018). Bioinformatics and genomic databases. Handb. Clin. Neurol. 147, 75–92. 10.1016/B978-0-444-63233-3.00007-5 29325629

[B7] Coulie P. G. Van Den Eynde B. J. Van Der Bruggen P. Boon T. J. N. R. C. (2014). Tumour antigens recognized by T lymphocytes: At the core of cancer immunotherapy. Nat. Rev. Cancer 14, 135–146. 10.1038/nrc3670 24457417

[B8] David C. J. Manley J. L. J. G. (2010). Alternative pre-mRNA splicing regulation in cancer: Pathways and programs unhinged. Genes Dev. 24, 2343–2364. 10.1101/gad.1973010 21041405 PMC2964746

[B9] Edin S. Wikberg M. L. Dahlin A. M. Rutegård J. Öberg Å. Oldenborg P.-A. (2012). The distribution of macrophages with a M1 or M2 phenotype in relation to prognosis and the molecular characteristics of colorectal cancer. LoS One 7 (10), e47045. 10.1371/journal.pone.0047045 PMC3471949 23077543

[B10] Emens L. a. J. I. R. O. I. (2006). Roadmap to a better therapeutic tumor vaccine. Int. Rev. Immunol. 25, 415–443. 10.1080/08830180600992423 17169782

[B11] Faghfuri E. Pourfarzi F. Faghfouri A. H. Abdoli Shadbad M. Hajiasgharzadeh K. Baradaran B. J. E. O. O. B. T. (2021). Recent developments of RNA-based vaccines in cancer immunotherapy. Expert Opin. Biol. Ther. 21, 201–218. 10.1080/14712598.2020.1815704 32842798

[B12] Fiedler K. Lazzaro S. Lutz J. Rauch S. Heidenreich R. J. C. S. I. C. G. T. (2016). mRNA cancer vaccines. Recent Results Cancer Res. 209, 61–85. 10.1007/978-3-319-42934-2_5 28101688

[B13] Galluzzi L. Vitale I. Warren S. Adjemian S. Agostinis P. Martinez A. B. (2020). Consensus guidelines for the definition, detection and interpretation of immunogenic cell death. J. Immunother. Cancer 8, e000337. 10.1136/jitc-2019-000337 32209603 PMC7064135

[B14] Gao G. Xie A. Huang S.-C. Zhou A. Zhang J. Herman A. M. (2011). Role of RBM25/LUC7L3 in abnormal cardiac sodium channel splicing regulation in human heart failure. Circulation 124, 1124–1131. 10.1161/CIRCULATIONAHA.111.044495 21859973 PMC3172047

[B15] Goodman A. M. Kato S. Bazhenova L. Patel S. P. Frampton G. M. Miller V. (2017). Tumor mutational burden as an independent predictor of response to immunotherapy in diverse cancers. Mol. Cancer Ther. 16, 2598–2608. 10.1158/1535-7163.MCT-17-0386 28835386 PMC5670009

[B16] Grammatikakis I. Zhang P. Panda A. C. Kim J. Maudsley S. Abdelmohsen K. (2016). Alternative splicing of neuronal differentiation factor TRF2 regulated by HNRNPH1/H2. Cell Rep. 15, 926–934. 10.1016/j.celrep.2016.03.080 27117401 PMC4856555

[B17] Gu X. Wang L. Boldrup L. Coates P. J. Fahraeus R. Sgaramella N. (2019). AP001056.1, A prognosis-related enhancer RNA in squamous cell carcinoma of the head and neck. Cancers (Basel) 11. 10.3390/cancers11030347 PMC6468641 30862109

[B18] Hao J. Jackson L. Calaluce R. Mcdaniel K. Dalkin B. L. Nagle R. B. J. T. a. J. O. P. (2001). Investigation into the mechanism of the loss of laminin 5 (alpha3beta3gamma2) expression in prostate cancer. Am. J. Pathol. 158, 1129–1135. 10.1016/s0002-9440(10)64060-6 11238061 PMC1850351

[B19] Hodges T. R. Ott M. Xiu J. Gatalica Z. Swensen J. Zhou S. (2017). Mutational burden, immune checkpoint expression, and mismatch repair in glioma: Implications for immune checkpoint immunotherapy. Neuro. Oncol. 19, 1047–1057. 10.1093/neuonc/nox026 28371827 PMC5570198

[B20] Huang S. H. Xu W. Waldron J. Siu L. Shen X. Tong L. (2015). Refining American Joint Committee on Cancer/Union for International Cancer Control TNM stage and prognostic groups for human papillomavirus-related oropharyngeal carcinomas. J. Clin. Oncol. 33, 836–845. 10.1200/JCO.2014.58.6412 25667292

[B21] Huang X. Tang T. Zhang G. Liang T. J. M. C. (2021a). Identification of tumor antigens and immune subtypes of cholangiocarcinoma for mRNA vaccine development. Mol. Cancer 20, 50–17. 10.1186/s12943-021-01342-6 33685460 PMC7938044

[B22] Huang X. Zhang G. Tang T. Liang T. J. M. C. (2021b). Identification of tumor antigens and immune subtypes of pancreatic adenocarcinoma for mRNA vaccine development. Mol. Cancer 20, 44–18. 10.1186/s12943-021-01310-0 33648511 PMC7917175

[B23] Katsuta E. Qi Q. Peng X. Hochwald S. N. Yan L. Takabe K. J. S. R. (2019). Pancreatic adenocarcinomas with mature blood vessels have better overall survival. Sci. Rep. 9, 1310–1311. 10.1038/s41598-018-37909-5 30718678 PMC6362082

[B24] Kelemen O. Convertini P. Zhang Z. Wen Y. Shen M. Falaleeva M. (2013). Function of alternative splicing. Gene 514, 1–30. 10.1016/j.gene.2012.07.083 22909801 PMC5632952

[B25] Kim M. S. Pinto S. M. Getnet D. Nirujogi R. S. Manda S. S. Chaerkady R. (2014). A draft map of the human proteome. Nature 509, 575–581. 10.1038/nature13302 24870542 PMC4403737

[B26] Kubler H. Scheel B. Gnad-Vogt U. Miller K. Schultze-Seemann W. Vom Dorp F. (2015). Self-adjuvanted mRNA vaccination in advanced prostate cancer patients: A first-in-man phase I/IIa study. J. Immunother. Cancer 3, 26. 10.1186/s40425-015-0068-y 26082837 PMC4468959

[B27] Kurebayashi Y. Ojima H. Tsujikawa H. Kubota N. Maehara J. Abe Y. (2018). Landscape of immune microenvironment in hepatocellular carcinoma and its additional impact on histological and molecular classification. Hepatology 68, 1025–1041. 10.1002/hep.29904 29603348

[B28] Leemans C. R. Snijders P. J. Brakenhoff R. H. J. N. R. C. (2018). The molecular landscape of head and neck cancer. Nat. Rev. Cancer 18, 269–282. 10.1038/nrc.2018.11 29497144

[B29] Li L.-Y. Yang Q. Jiang Y.-Y. Yang W. Jiang Y. Li X. (2021). Interplay and cooperation between SREBF1 and master transcription factors regulate lipid metabolism and tumor-promoting pathways in squamous cancer. Nat. Commun. 12, 4362. 10.1038/s41467-021-24656-x 34272396 PMC8285542

[B30] Litchfield K. Reading J. L. Lim E. L. Xu H. Liu P. Al-Bakir M. (2020). Escape from nonsense-mediated decay associates with anti-tumor immunogenicity. Nat. Commun. 11, 3800–3811. 10.1038/s41467-020-17526-5 32733040 PMC7393139

[B31] Mcgranahan N. Furness A. J. Rosenthal R. Ramskov S. Lyngaa R. Saini S. K. (2016). Clonal neoantigens elicit T cell immunoreactivity and sensitivity to immune checkpoint blockade. Science 351, 1463–1469. 10.1126/science.aaf1490 26940869 PMC4984254

[B32] Mcnamara M. A. Nair S. K. Holl E. K. J. J. O. I. R. (2015). RNA-based vaccines in cancer immunotherapy. J. Immunol. 2015, 794528. 10.1155/2015/794528 PMC4668311 26665011

[B33] Meng H.-X. Yang X.-X. Liu R.-Q. Bao J.-J. Hou Y.-J. Sun J. (2020). The relationship between human papillomavirus, OFD1 and primary ciliogenesis in the progression of oropharyngeal cancer: A retrospective cohort study. Pharmgenomics. Pers. Med. 13, 633–644. 10.2147/PGPM.S271735 33244255 PMC7685095

[B34] Miao L. Zhang Y. Huang L. (2021). mRNA vaccine for cancer immunotherapy. Mol. Cancer 20, 41. 10.1186/s12943-021-01335-5 33632261 PMC7905014

[B35] Nogueira G. Fernandes R. García-Moreno J. F. Romão L. J. M. C. (2021). Nonsense-mediated RNA decay and its bipolar function in cancer. Mol. Cancer 20, 72–19. 10.1186/s12943-021-01364-0 33926465 PMC8082775

[B36] Noy R. Pollard J. W. J. I. (2014). Tumor-associated macrophages: From mechanisms to therapy. Immunity 41, 49–61. 10.1016/j.immuni.2014.06.010 25035953 PMC4137410

[B37] Pardi N. Hogan M. J. Weissman D. J. C. O. I. I. (2020). Recent advances in mRNA vaccine technology. Curr. Opin. Immunol. 65, 14–20. 10.1016/j.coi.2020.01.008 32244193

[B38] Postow M. A. Sidlow R. Hellmann M. D. (2018). Immune-related adverse events associated with immune checkpoint blockade. N. Engl. J. Med. 378, 158–168. 10.1056/NEJMra1703481 29320654

[B39] Ribas A. Wolchok J. D. J. S. (2018). Cancer immunotherapy using checkpoint blockade. Science 359, 1350–1355. 10.1126/science.aar4060 29567705 PMC7391259

[B40] Rizvi N. A. Hellmann M. D. Snyder A. Kvistborg P. Makarov V. Havel J. J. (2015). Cancer immunology. Mutational landscape determines sensitivity to PD-1 blockade in non-small cell lung cancer. Science 348, 124–128. 10.1126/science.aaa1348 25765070 PMC4993154

[B41] Rooney M. S. Shukla S. A. Wu C. J. Getz G. Hacohen N. (2015). Molecular and genetic properties of tumors associated with local immune cytolytic activity. Cell 160, 48–61. 10.1016/j.cell.2014.12.033 25594174 PMC4856474

[B42] Sato E. Olson S. H. Ahn J. Bundy B. Nishikawa H. Qian F. (2005). Intraepithelial CD8+ tumor-infiltrating lymphocytes and a high CD8+/regulatory T cell ratio are associated with favorable prognosis in ovarian cancer. Proc. Natl. Acad. Sci. U. S. A. 102, 18538–18543. 10.1073/pnas.0509182102 16344461 PMC1311741

[B43] Sayour E. J. Mendez-Gomez H. R. Mitchell D. a. J. I. J. O. M. S. (2018). Cancer vaccine immunotherapy with RNA-loaded liposomes. Int. J. Mol. Sci. 19, 2890. 10.3390/ijms19102890 30249040 PMC6213933

[B44] Sha D. Jin Z. Budczies J. Kluck K. Stenzinger A. Sinicrope F. a. J. C. D. (2020). Tumor mutational burden as a predictive biomarker in solid tumors. Cancer Discov. 10, 1808–1825. 10.1158/2159-8290.CD-20-0522 33139244 PMC7710563

[B45] Shahnazari M. Samadi P. Pourjafar M. Jalali A. J. I. I. (2020). Therapeutic vaccines for colorectal cancer: The progress and future prospect. Int. Immunopharmacol. 88, 106944. 10.1016/j.intimp.2020.106944 33182032

[B46] Shen Y. Zhang L. Piao S. Li L. Li J. Xia Y. (2020). NUDT1: A potential independent predictor for the prognosis of patients with oral squamous cell carcinoma. J. Oral Pathol. Med. 49, 210–218. 10.1111/jop.12974 31732994

[B47] Sullenger B. A. Nair S. J. S. (2016). From the RNA world to the clinic. Science 352, 1417–1420. 10.1126/science.aad8709 27313039 PMC6035743

[B48] Sung H. Ferlay J. Siegel R. L. Laversanne M. Soerjomataram I. Jemal A. (2021). Global cancer statistics 2020: GLOBOCAN estimates of incidence and mortality worldwide for 36 cancers in 185 countries. Ca. Cancer J. Clin. 71, 209–249. 10.3322/caac.21660 33538338

[B49] Thorsson V. Gibbs D. L. Brown S. D. Wolf D. Bortone D. S. Yang T.-H. O. (2018). The immune landscape of cancer. Immunity 48, 812–830. 10.1016/j.immuni.2018.03.023 29628290 PMC5982584

[B50] Turunen J. J. Verma B. Nyman T. A. Frilander M. J. J. R. (2013). HnRNPH1/H2, U1 snRNP, and U11 snRNP cooperate to regulate the stability of the U11-48K pre-mRNA. RNA 19, 380–389. 10.1261/rna.036715.112 23335637 PMC3677248

[B51] Valk-Lingbeek M. E. Bruggeman S. W. Van Lohuizen M. J. C. (2004). Stem cells and cancer: The polycomb connection. Cell 118, 409–418. 10.1016/j.cell.2004.08.005 15315754

[B52] Van Nuffel A. M. Wilgenhof S. Thielemans K. Bonehill A. (2012). Overcoming HLA restriction in clinical trials: Immune monitoring of mRNA-loaded DC therapy. Oncoimmunology 1, 1392–1394. 10.4161/onci.20926 23243604 PMC3518513

[B54] Venables J. P. J. C. R. (2004). Aberrant and alternative splicing in cancer. Cancer Res. 64, 7647–7654. 10.1158/0008-5472.CAN-04-1910 15520162

[B55] Vermorken J. B. Peyrade F. Krauss J. Mesia R. Remenar E. Gauler T. C. (2014). Cisplatin, 5-fluorouracil, and cetuximab (PFE) with or without cilengitide in recurrent/metastatic squamous cell carcinoma of the head and neck: Results of the randomized phase I/II ADVANTAGE trial (phase II part). Ann. Oncol. 25, 682–688. 10.1093/annonc/mdu003 24567516 PMC3933250

[B56] Xing L. Zhang X. Tong D. J. D. Biology C. (2019). Systematic profile analysis of prognostic alternative messenger RNA splicing signatures and splicing factors in head and neck squamous cell carcinoma. DNA Cell Biol. 38, 627–638. 10.1089/dna.2019.4644 31025877

[B57] Ye L. Wang L. Yang J. Hu P. Zhang C. Tong S. (2021). Identification of tumor antigens and immune landscape in glioblastoma for mRNA vaccine development. Front. Genet. 12, 701065. 10.3389/fgene.2021.701065 34527020 PMC8435740

[B58] Zhang Y. Qian J. Gu C. Yang Y. (2021). Alternative splicing and cancer: A systematic review. Signal Transduct. Target. Ther. 6, 78. 10.1038/s41392-021-00486-7 33623018 PMC7902610

[B59] Zhao X. Si S. Li X. Sun W. Cui L. J. J. O. C. (2020). Identification and validation of an alternative splicing-based prognostic signature for head and neck squamous cell carcinoma. J. Cancer 11, 4571–4580. 10.7150/jca.44746 32489474 PMC7255372

[B60] Zhong H. Liu S. Cao F. Zhao Y. Zhou J. Tang F. (2021). Dissecting tumor antigens and immune subtypes of glioma to develop mRNA vaccine. Front. Immunol. 12, 709986. 10.3389/fimmu.2021.709986 34512630 PMC8429949

[B61] Zhong Q. Fang J. Huang Z. Yang Y. Lian M. Liu H. (2018). A response prediction model for taxane, cisplatin, and 5-fluorouracil chemotherapy in hypopharyngeal carcinoma. Sci. Rep. 8, 12675. 10.1038/s41598-018-31027-y 30139993 PMC6107664

