# Supplementary material for: Identification of tumor antigens and immune subtypes in head and neck squamous cell carcinoma for mRNA vaccine development
Source: Front Cell Dev Biol. 2022 Nov 17;10:1064754. doi: 10.3389/fcell.2022.1064754 (PMC9714632; doi:10.3389/fcell.2022.1064754)
Supplement: Supplementary file 1 [file DataSheet1.docx]

**Supplementary Information**

**Identification of tumor antigens and immune subtypes in head and neck squamous cell carcinoma for mRNA vaccine development**

Yan Chen ^1, †^, Ning Jiang ^2, †^, Meihua Chen ^1, *^, Baiyan Sui ^3, *^, Xin Liu ^3, *^

^1^ Department of Periodontology, Shanghai Stomatological Hospital & School of Stomatology, Fudan University; Shanghai Key Laboratory of Craniomaxillofacial Development and Diseases, Fudan University, Shanghai, China

^2^ Department of Oral and Craniomaxillofacial Science, Shanghai Key Laboratory of Stomatology, College of Stomatology, Ninth People’s Hospital, Shanghai Jiao Tong University School of Medicine, Shanghai, China

^3^ Department of Dental Materials, Shanghai Key Laboratory of Stomatology, Shanghai Biomaterials Research & Testing Center, Shanghai Ninth People’s Hospital, Shanghai Jiao Tong University School of Medicine; College of Stomatology, Shanghai Jiao Tong University; National Center for Stomatology; National Clinical Research Center for Oral Diseases, Shanghai, China

*** *Correspondence:***

*Meihua Chen, chenmeihua@fudan.edu.cn*

*Baiyan Sui,* [*sby3808@126.com*](mailto:sby3808@126.com)

*Xin Liu,* [*liuxin0556@163.com*](mailto:liuxin0556@163.com)

†These authors contributed equally to this work and share the first authorship.

**Supplementary Tables**

**Table S1** Types of 537 TCGA-HNSCC samples

|  | type | number |
| --- | --- | --- |
| type | normal | 44 |
|  | cancer | 493 |

**Table S2** Clinical information of 493 TCGA-HNSCC cancer samples

|  | type | number |
| --- | --- | --- |
| OS | Alive | 280 |
|  | Dead | 213 |
| TP53_mutation | NO | 143 |
|  | YES | 350 |
| alcohol_history | NA | 11 |
|  | NO | 152 |
|  | YES | 330 |
| Stage | NA | 13 |
|  | I | 19 |
|  | II | 93 |
|  | III | 101 |
|  | IV | 267 |
| Grade | NA | 19 |
|  | G1 | 61 |
|  | G2 | 294 |
|  | G3 | 117 |
|  | G4 | 2 |
| clinical_T | NA | 14 |
|  | T1 | 32 |
|  | T2 | 142 |
|  | T3 | 129 |
|  | T4 | 176 |
| Gender | Female | 131 |
|  | Male | 362 |
| radiation_therapy | NA | 105 |
|  | NO | 133 |
|  | YES | 255 |

**Supplementary Figures**


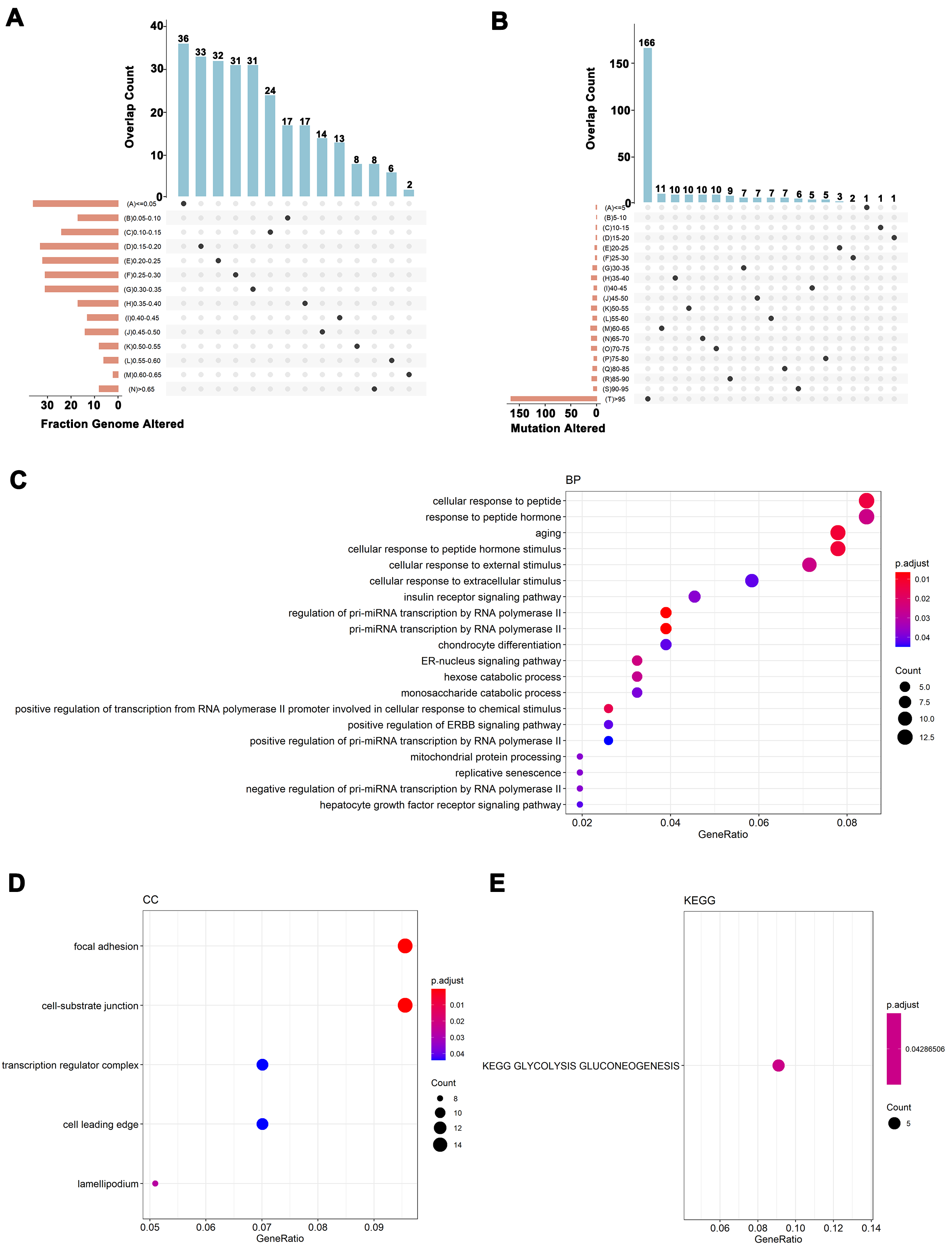


**Figure S1. Identification of potential antigen candidate genes.** (A) Samples in altered genome fraction. (B) Samples in mutation count groups. (C-E) Functional enrichment analysis of potential antigen candidate genes for (C) BP, (D) CC, and (E) KEGG.

**
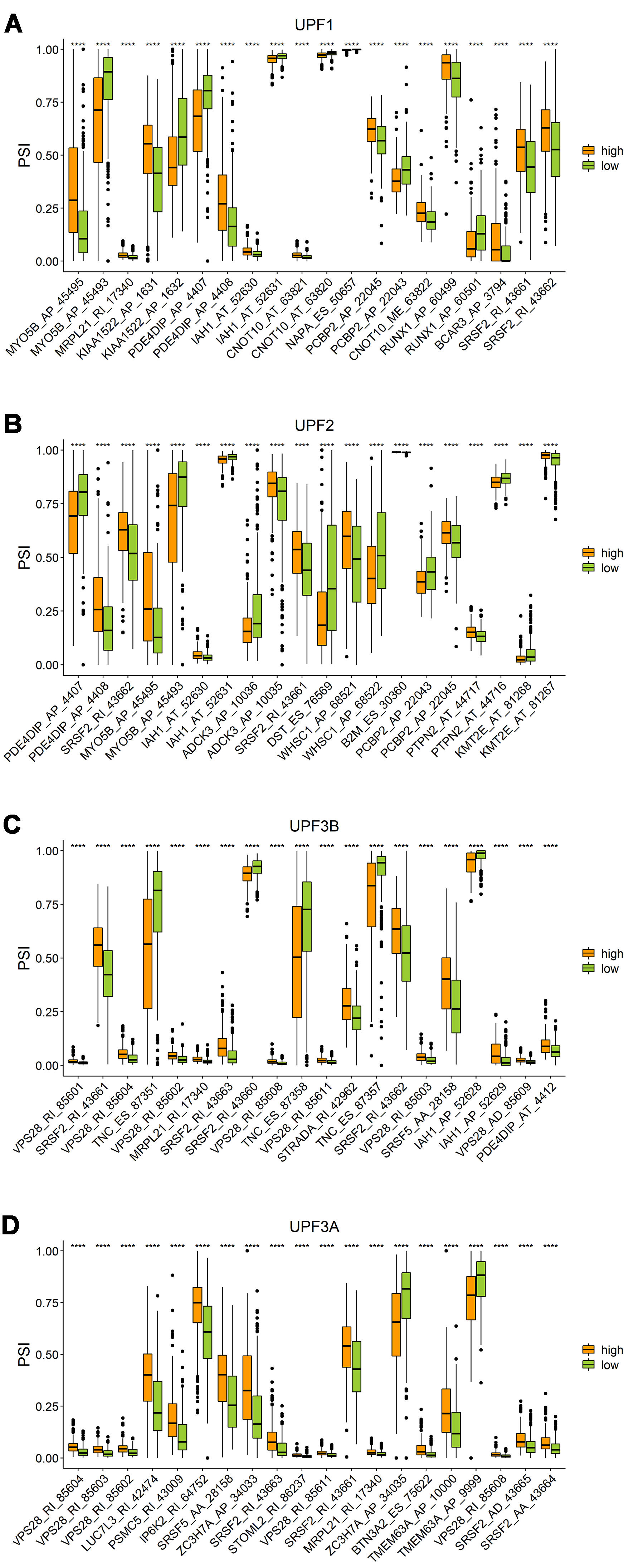
**

**Figure S2. The top 20 most significantly differentially distributed alternative splicing events were ranked according to the p-value of the t-test.** (A) UPF1, (B) UPF2, (C) UPF3A, and (D) UPF3B. **** p ≤ 0.0001.


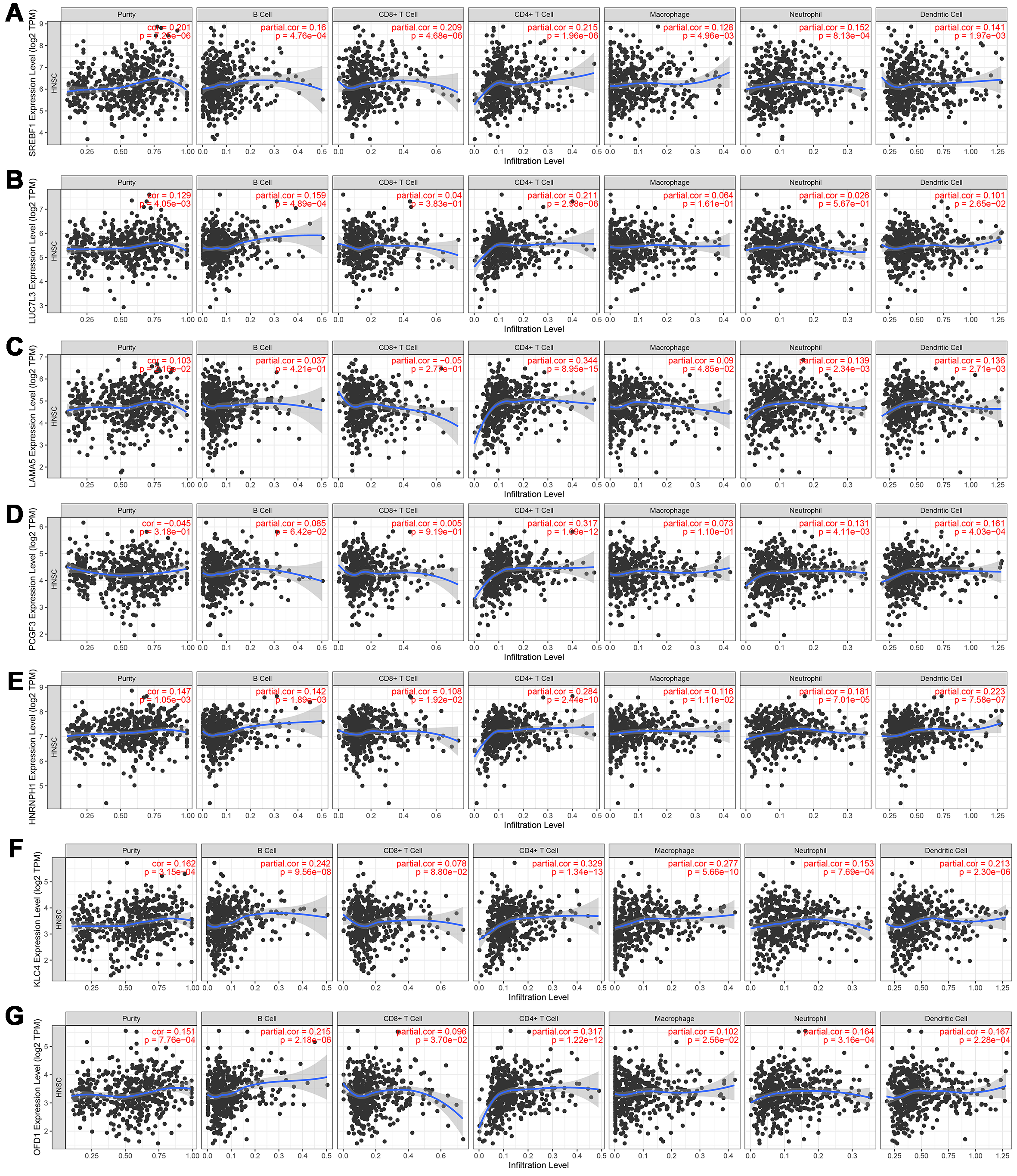


**Figure S3. Correlation of potential antigenic candidate genes with infiltration of APCs.** Correlations between gene expression and tumor purity, B cell, CD8^+^ T cell, CD4^+^ T cell, Macrophage, Neutrophil, and Dendritic infiltration ratios were demonstrated, respectively. (A) SREBF1, (B) LUC7L3, (C) LAMA5, (D) PCGF3, (E) HNRNPH1, (F) KLC4, and (G) OFD1.


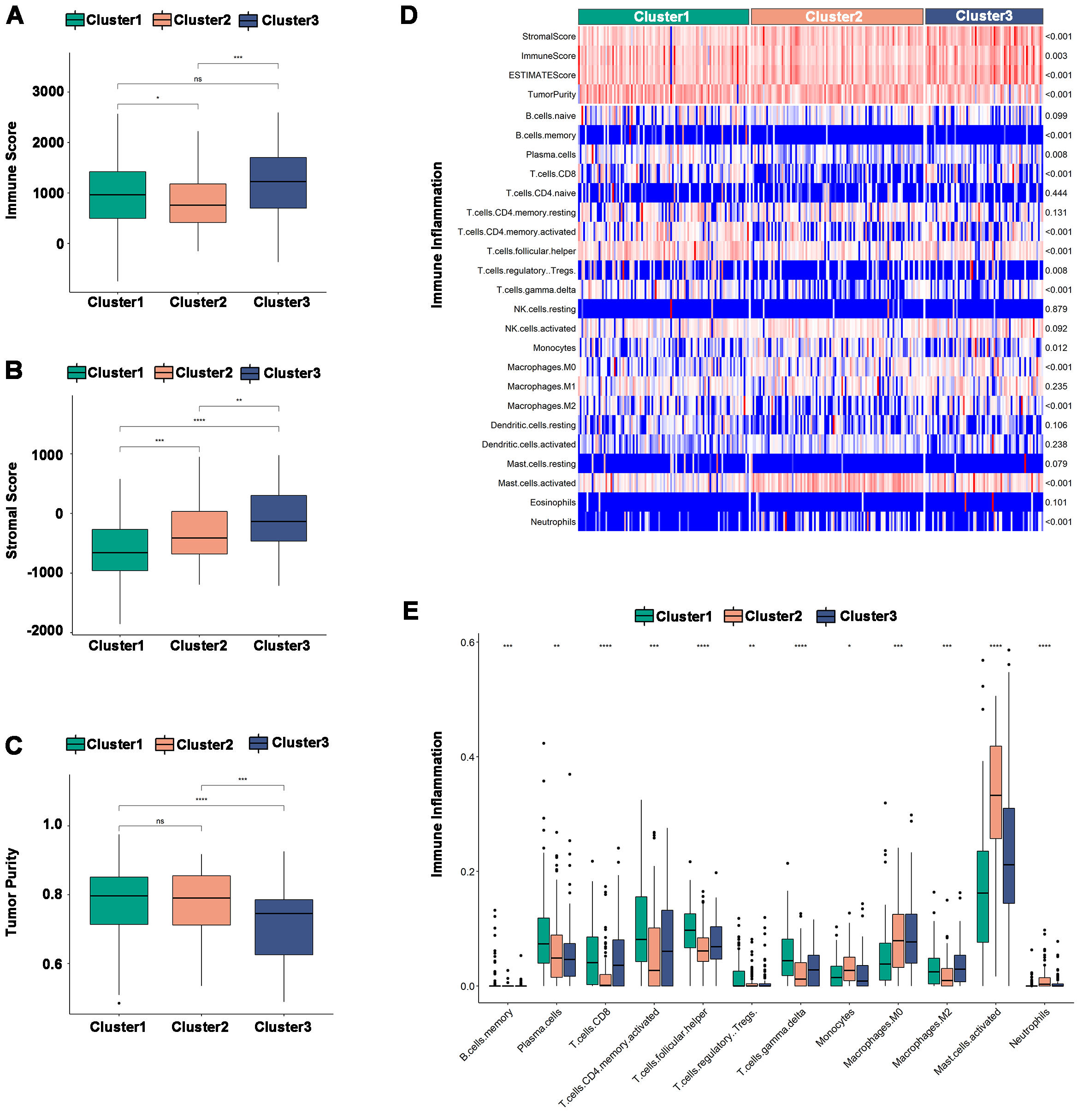


**Figure S4. Cellular and molecular characteristics of immune subtypes in the validation set (GEO).** (A-C) Differential distribution of (A) immune scores, (B) stromal scores, and (C) tumor purity among the three immune subtypes. (D) Heatmap of immune cell infiltration ratio. (E) Differential distribution of immune cell infiltration ratio. * p ≤ 0.05, ** p ≤ 0.01, *** p ≤ 0.001, **** p ≤ 0.0001, and ns, non-significant.

**
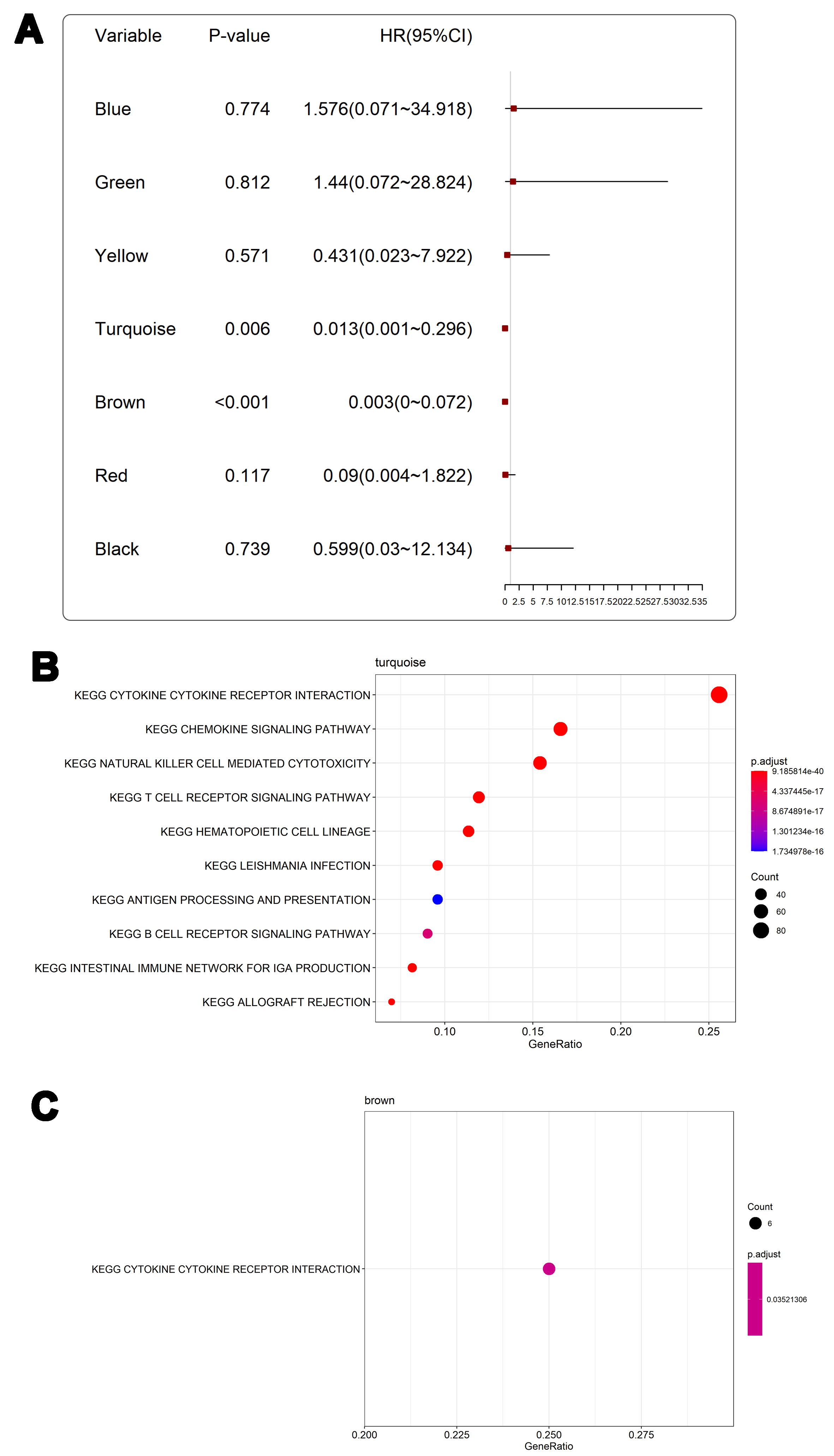
**

**Figure S5. Prognostic module identification and functional annotation.** (A) One-way Cox regression analysis of seven modules. (B) Enrichment analysis of the Turquoise module. (C) Enrichment analysis of Brown module.


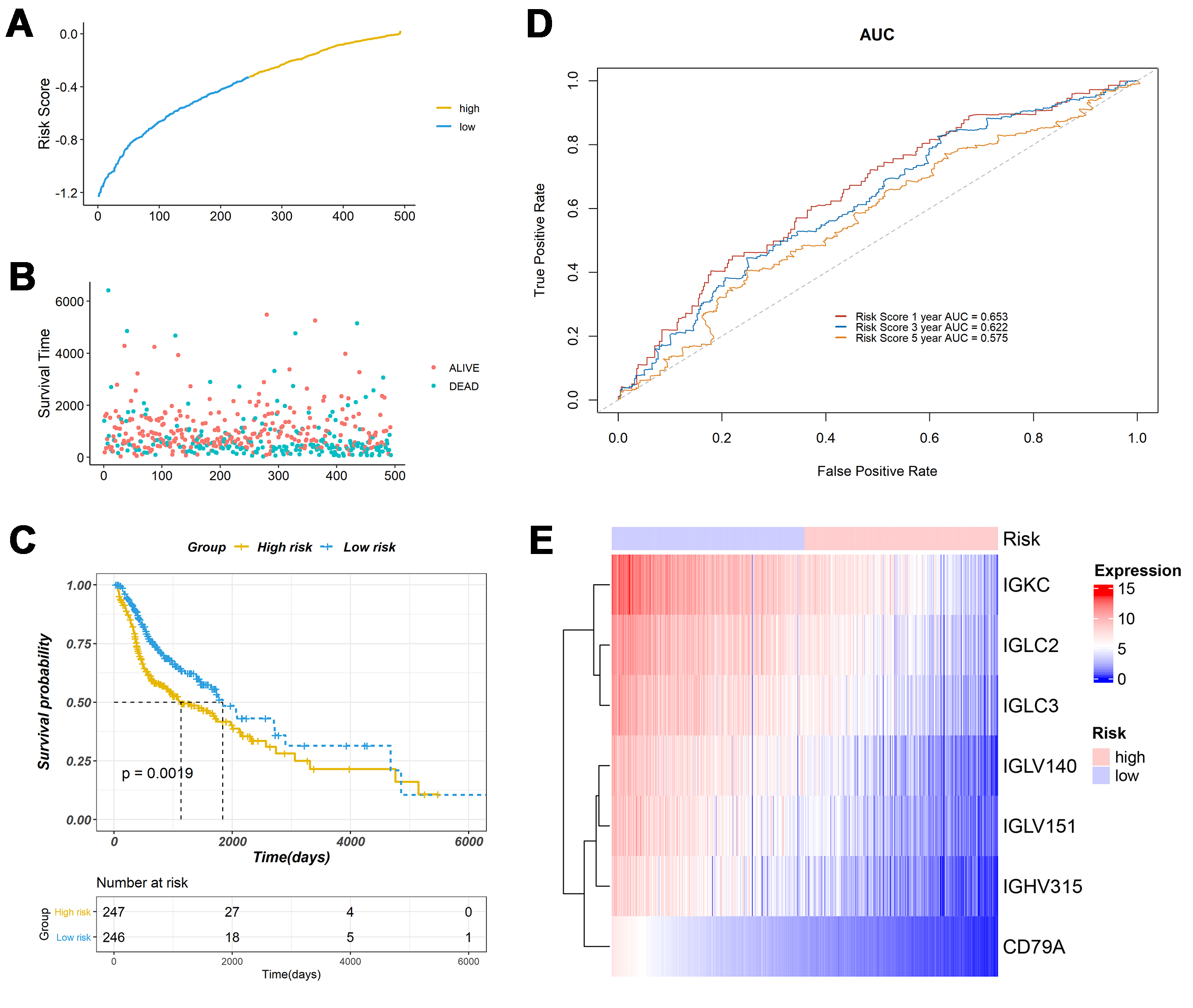


**Figure S6. The risk model based on biomarker genes.** (A) Risk scores of high and low risk groups. (B) Survival status of high and low risk groups. (C) KM curves of scores. (D) Assessment of model predictive efficacy. (E) Heatmap of the expression of biomarker genes.
